# Local structure elucidation of tungsten-substituted vanadium dioxide (V$_{1-x}$W$_x$O$_2$)

**DOI:** 10.1038/s41598-022-18575-0

**Published:** 2022-08-30

**Authors:** Catrina E. Wilson, Amanda E. Gibson, Paul M. Cuillier, Cheng-Han Li, Patrice H. N. Crosby, Edward B. Trigg, Stan Najmr, Christopher B. Murray, Joerg R. Jinschek, Vicky Doan-Nguyen

**Affiliations:** 1 Materials Science and Engineering, Ohio State University, Columbus, OH 43212 USA; 2 Chemistry, Ohio State University, Columbus, OH 43212 USA; 3 Materials & Manufacturing Directorate, Air Force Research Laboratory, Wright-Patterson Air Force Base, OH 45433 USA; 4 Chemistry, University of Pennsylvania, Philadelphia, PA 19143 USA; 5 Present Address: Materials Science and Engineering, University of Pennsylvania, Philadelphia, PA 19143 USA; 6 Present Address: Human Centered Design, Cornell University, Ithaca, NY 14853 USA; 7 Present Address: Battelle Memorial Institute, Columbus, OH 43201 USA; 8 Present Address: DTU Nanolab, Technical University of Denmark, 2800 Kongens Lyngby, Denmark

**Keywords:** Energy science and technology, Engineering, Materials science

## Abstract

Initially, vanadium dioxide seems to be an ideal first-order phase transition case study due to its deceptively simple structure and composition, but upon closer inspection there are nuances to the driving mechanism of the metal-insulator transition (MIT) that are still unexplained. In this study, a local structure analysis across a bulk powder tungsten-substitution series is utilized to tease out the nuances of this first-order phase transition. A comparison of the average structure to the local structure using synchrotron x-ray diffraction and total scattering pair-distribution function methods, respectively, is discussed as well as comparison to bright field transmission electron microscopy imaging through a similar temperature-series as the local structure characterization. Extended x-ray absorption fine structure fitting of thin film data across the substitution-series is also presented and compared to bulk. Machine learning technique, non-negative matrix factorization, is applied to analyze the total scattering data. The bulk MIT is probed through magnetic susceptibility as well as differential scanning calorimetry. The findings indicate the local transition temperature ($T_c$) is less than the average $T_c$ supporting the Peierls-Mott MIT mechanism, and demonstrate that in bulk powder and thin-films, increasing tungsten-substitution instigates local V-oxidation through the phase pathway VO$_2\, \rightarrow$ V$_6$O$_{13} \, \rightarrow$ V$_2$O$_5$.

## Introduction

**Figure 1:**
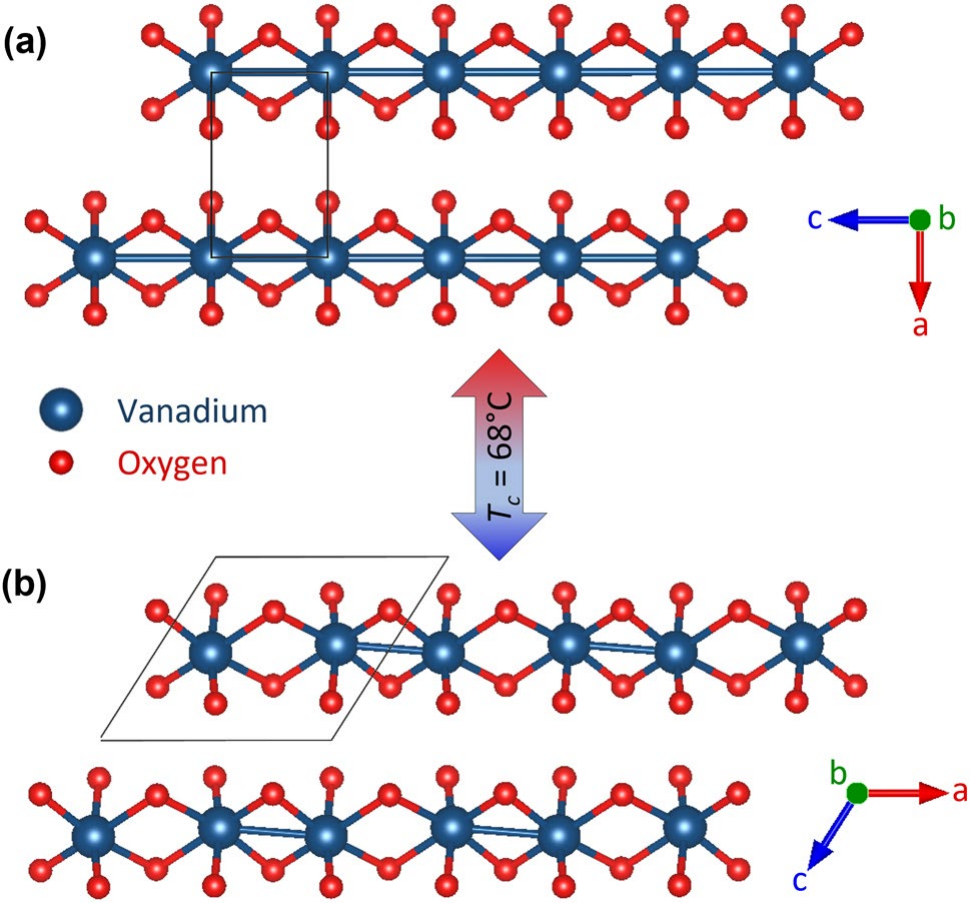
Structural changes across the MIT illustrating V–V dimerization changes accompanying structure transformation from (**a**) $P4_2/mnm$ to (**b**) $P2_1/c$ upon cooling with $T_c$ occuring at 68 °C.

Vanadium dioxide (VO$_2$) undergoes a fully reversible metal-insulator transition (MIT) that drastically changes the structure and properties despite slight compositional^1^, thermal^2,3^, electrical^4,5^, or optical^6,7^ perturbations. Across the MIT ($T_c = {68}$ °C), VO$_2$’s paramagnetic moment increases by nearly an order of magnitude^3^, the electrical conductivity increases by 4–5 orders of magnitude^8,9^ depending on oxygen-vacancy concentration instigating V reduction^10^, and infrared transmittance decreases by almost an order of magnitude^11,12^. The MIT is concomitant with a structural phase transformation (SPT) from the low-temperature monoclinic structure^13^, $P2_1/c$, to the high-temperature tetragonal structure^14^, $P4_2/mnm$ (Fig. 1). Due to the reversible property changes, VO$_2$ is suitable for switching applications such as memory storage^15–17^, smart windows^18–21^, infrared detection^22^ or evasion^23^, photocatalysis^19,24^, thermal energy storage^25^, and radio-frequency modulation^26,27^. The remaining challenges facing the commercial implementation of VO$_2$ include a complex or time-consuming synthesis, a relatively high $T_c$, and an unresolved MIT mechanism.

Most VO$_2$ applications hinge on a thin-film morphology^28^ but the inherent strain-effects in thin films could induce phase transformations outside of the expected phases^29^ obfuscating the MIT mechanism. Traditional bulk syntheses require a multi-day anneal to achieve phase purity^30^. Novel synthesis development is challenging because of the many stable oxidation states^31^ and structural polymorphs^32^ within the V–O system. Microwave-facilitated solid-state syntheses have recently been demonstrated^33,34^. A microwave-facilitated W$_x$V$_{1-x}$O$_2$ powder synthesis has been established^35^ decreasing the synthesis time from 11 days to 45 mins while maintaining phase purity, morphology, and structural selectivity.

Modulation of VO$_2$’s MIT temperature is essential due to the wide-variety of potential applications. Fortunately, tuning of the MIT is accessible through strain engineering^36^ as well as cation-substitution with a variety of elements such as Al^37–39^, Sc^40^, Ti^37,41–44^, Cr^37,45–49^, Fe^50–52^, Ga^53^, Ge^54^, As^55^, Nb^37,56,57^, Mo^44,58–60^, Ru^55^, Ta^1^, W^3,37,41,61–63^, Re^44^, Os^55^, and Ir^55^. As well as anion-substitution with F^63–65^. Cation-substitution of Al, Cr, Fe, Ga, and Ge raises the transition temperature; whereas, cation-substitution of Sc, Ti, Nb, Mo, and W as well as anion-substitution of F, lowers the $T_c$.

Why some elements increase $T_c$ while others decrease $T_c$ is still unresolved and hinders prediction upon new substitutions. For example, Sc was expected to most effectively decrease the MIT from ab initio calculations^66^ but a recent study^40^ has found that the bulk $T_c$ does not change appreciably even after 6.25 at% Sc-substitution. A unified examination of substitutions that increase, decrease, and negligibly change $T_c$ is needed to advance VO$_2$ applications.

Even after decades long debate^1,2,67^, VO$_2$’s MIT mechanism remains unresolved. Ab initio calculations have not supported the singular Mott-Hubbard^68,69^ (electronic correlations) or Peierls^70–72^ (electron-phonon interaction) hypotheses. The Peierls instability was unsupported by the local density approximation (LDA) calculation which correctly predicts the monoclinic phase as the lowest energy^70,71^ but it cannot capture the band gap in the insulating ground state^72^. LDA plus static on-site repulsion (LDA+U) calculations employed to test the Mott-Hubbard hypothesis, predicted correctly the low-temperature insulating state, but did not predict the high-temperature metallic phase^68,69^. A non-local cluster cellular dynamical mean-field theory (c-DMFT) model was implemented^73,74^ and correctly described low-temperature VO$_2$ as an insulator with a band gap in agreement with experimental data. This technique demonstrated that the V–V dimerization is a bonding of the $d_{xy}$ and $d_{xz}$ orbitals along the rutile *c*-axis, with one electron in each orbital, generating a Mott instability. It was also found that the Peierls state is robust even under reduction of the long-range crystallographic disorder and local impurities. This transition mechanism has been identified as a Peierls-assisted-orbital-selective-Mott transition or Peierls-Mott transition^73,74^. This c-DMFT model supports recent in-situ studies that have uncovered metallic “nanopuddles” which form prior to the expected MIT^75,76^.

In this work, we reveal distinctions between the local structure SPT and the average structure SPT before the expected MIT $T_c$. Pair distribution functions (PDF) and transmission electron microscopy (TEM) bright field (BF) imaging were compared to synchrotron x-ray diffraction (XRD) in a systematic and quantitative analysis to identify local and average structures throughout the phase transformation. PDFs’s were analyzed through conventional fitting methods^77^ as well as machine learning technique, non-negative matrix factorization (NMF). The local and average SPT temperature gathered from PDF, TEM, and XRD was then compared to the bulk MIT temperature probed through differential scanning calorimetry (DSC) and magnetization data. In each case, it was found that the local SPT temperature varies depending on tungsten (W) substitution concentration and precedes the average SPT temperature and the MIT $T_c$. The local SPT temperature being lower than the average SPT temperature was attributed to localized strain. The local SPT temperature being lower than the bulk MIT $T_c$ supports the Peierls-Mott hypothesis of MIT origin in the correlated W$_x$V$_{1-x}$O$_2$. These two observations are similar to the “nanopuddle” observation^75^. The final conclusion drawn from the presented PDF as well as extended x-ray fine structure (EXAFS) refinement is that W-substitution drives V oxidation from VO$_2$ to V$_6$O$_{13}$ (*C*2/*m*) to V$_2$O$_5$ (*Pnma*) which has not yet been established in the community. The V oxidation as well as the localized nucleation of the $P4_2/mnm$ phase prior to the MIT, decreases the distinction of the switching mechanism due to the diminished difference in property change across the MIT.

## Results and discussion

### W-substitution induces local SPT prior to average SPT

Phase purity was validated through Rietveld refinement of synchrotron powder XRD across an array of temperatures (Figs. S1 and S2). All elemental compositions were determined through inductively coupled plasma optical emission spectroscopy where atomic concentrations of W and V were independently established prior to Rietveld analysis (Table S1). This allowed for greater precision during the Rietveld refinement of the site occupancies. The refinement analysis accounted for all peaks present with no impurity peaks emerging. All goodness-of-fit parameters, $R_{wp}$ were below 10%, demonstrating the integrity of the Rietveld refinement to the designated phase^78^.

At room-temperature, compositions below 2.5 at% W fit best to the $P2_1/c$ phase, compositions above 3.6 at% fit best to the $P4_2/mnm$ phase, and compositions 2.5 at% and 3.6 at% fit best to a co-refinement to both phases through comparison of goodness-of-fit values. While compositions between 0.8 at% and 6.3 at% were best represented with a co-refinement of both phases with varying phase fractions (Tables S2–S9). When $P4_2/mnm$ becomes the majority phase fraction at 3.6 at% W-substitution, the fit residuals decreased by approximately half due to the increased symmetry of the tetragonal phase compared to the monoclinic phase. However, increasing W-substitution beyond 3.6 at% up to 15 at% gradually increased the fit residuals (0.0632%/at%) due to increasing disorder of the V position within the lattice. This disorder is attributed to ionic radii differences, assuming identical coordination numbers, between W and V.

The change in lattice parameters $a, b, c, \beta$, cell volume, and *c*/*a* were followed as a function of W-substitution amount (Fig. S3). Linear regressions determined the average rate of expansion in each lattice parameter and the overall impact on the *c*/*a* ratio. Linear regressions were performed to compositions whose majority phase was $P2_1/c$, at% < 3.6, and whose majority phase was $P4_2/mnm$, at% $> 0.8$, excluding outliers characterized by increased error due to small (< 0.1) phase fraction. All linear regressions were of reasonable fidelity with all fits to the $P4_2/mnm$ data producing $R^2$ values greater than 95%, and all fits to the $P2_1/c$ data produced $R^2$ values greater than 80% due to the limited sample size (Table S10).

The linear regressions for W-substitution amounts below 3.6 at% indicate that the lattice expands twice as fast in the *a*-direction as the *b*-direction, and five times as fast as the *c*-direction. The transformation matrix between the two phases dictates that the *a*-axis of the monoclinic lattice becomes the *c*-axis of the tetragonal lattice.

$$\begin{aligned} \begin{pmatrix} a \\ b \\ c \\ \end{pmatrix}_{P2_1/c} = \begin{pmatrix} a \\ b \\ c \\ \end{pmatrix}_{P4_2/mnm} \begin{pmatrix} 0 &{} 0 &{} -2 \\ 0 &{} 1 &{} 0\\ 1 &{} 0 &{} 1 \\ \end{pmatrix} \end{aligned}$$

Once this occurs and the structure is transitioned to the tetragonal phase, the *a*-direction expansion decreases by a factor of three. The slower lattice expansion after phase transformation is due to the 12.9% ionic radii difference between V (0.58 Å) and W (0.66 Å), assuming they are both in the $4+$-oxidation state in an 6-coordinate environment^79^.

While in the monoclinic phase, the lattice expansion leads to a decrease in the *c*/*a* parameter and an increase in *c*/*a*, when in the tetragonal phase. The structural transition occurs when the *c*/*a* ratio reaches 0.62663(4), in good agreement with the 0.625 value reported previously^80^ for the structural transformation in the rutile phase. As W is introduced into the structure, V−V bonding strength is increased as the atoms are driven closer together due to the larger W-ions. This continues until the structure fully transitions into tetragonal with continuous metal-metal bonding along the rutile *c*-axis or monoclinic *a*-axis. Average structure data will now be compared to similar local structure information.

”Boxcar” refinements^81^ were performed on synchrotron total x-ray scattering PDF where varying *r*-ranges were fit separately, to create distinct regions of ”local” structure, ”intermediate” structure, and ”long-range” structure (Fig. S4). The refinements produced goodness-of-fit parameters below 10% supporting the phase purity conclusion drawn from the previous Rietveld refinements. Due to the anisotropic nature of the monoclinic structure ($a \ne b \ne c$), three boxcar fits best represented the data if there was any phase fraction of monoclinic structure in either the PDF or the XRD (Tables S11–S18). However, once the structure was fully tetragonal the best fits were achieved with two boxcars corresponding to a decrease in degrees of freedom with $a = b \ne c$. These refinements produced local structure unit cell information that was directly comparable to the average structure unit cell information from XRD.

The data was grouped into two boxcars labeled as the short-range and long-range data corresponding approximately to *r*-values lying between 0–19.2 Å and 19.2–30 Å, respectively. The *r*-ranges of each boxcar does depend on the dataset however, and more precise ranges can be found in Fig. S4). Lattice parameters were followed similarly to the Rietveld refinements but for each *r*-range (Fig. S5). Similarly to the average structure unit cell, there is an increase of the *a*-axis in both the short-range and long-range, when the structure is mostly monoclinic (Table S19). The hypothesis that W introduction drives V atoms closer together and increases the metal-metal bonding is supported by the almost 5.5 times increase in the *a*-axis expansion when comparing the local structure to the average structure. This corresponds to a local decrease in *c*/*a* of − 0.003(2) at%$^{-1}$ (Table S20) compared to an increase of 0.00027(1) at%$^{-1}$ (Table S21) in the long range with the average structure lying between the two at − 0.00071(6) at%. Most of the recent W-substitution literature^23,61,82–93^ uses average structure information to draw conclusions from, but it has been demonstrated that locally the structure changes much more drastically than the average structure indicates.

Approximate strain energies were calculated from the rates of lattice parameter change across the W-substitution series. Energies were derived from typical strain energy^94^, $\Delta G_S \approx 4\mu \delta ^2 V$, where $\mu$ is the shear modulus, $\delta$ is the unconstrained misfit, and *V* is the volume of the inclusion atom. If the following assumptions are made: $\mu$ correlates with *B*, $\delta$ correlates with $\frac{\Delta V}{V}$, and the inclusion atom is W therefore $V = V_W$, where *B* is the bulk modulus of VO$_2$^66,95^, and $\Delta V$ is the change in the lattice volume upon W-substitution, with *V* is the volume of the unsubstituted lattice. The strain energy equation then becomes:

1 $$\begin{aligned} \Delta G_S = 4 B (\frac{\Delta V}{V_0})^2 V_W \end{aligned}$$

**Figure 2:**
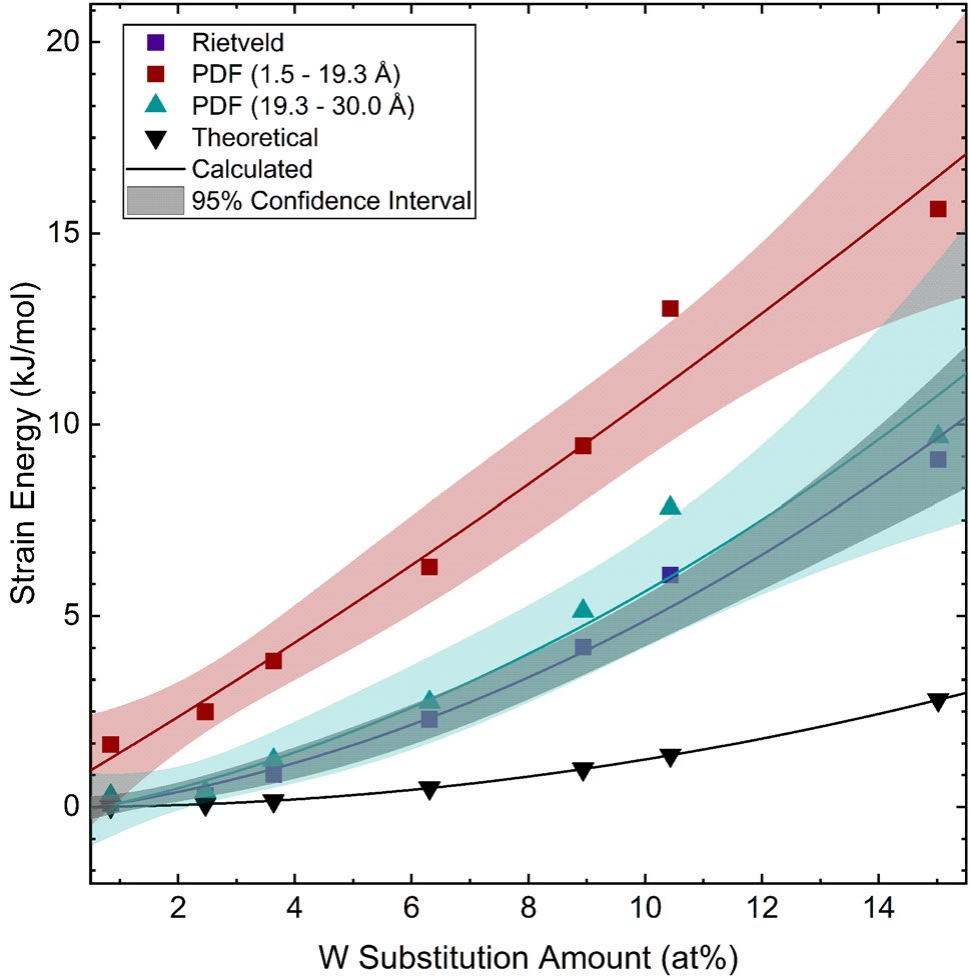
Local approximate strain is greater than long-range strain from PDF and Rietveld refinements. Both local and long-range strain are greater than theoretical strain calculated from effective ionic radii differences between W and V.

Theoretical strain energies were calculated assuming that the volumetric lattice expansion depends on differences in the atomic radii of W and V, as well as the W-substitution amount such that $\Delta V = V_0 \frac{V_W}{V_V}$x where $V_0$ is the unsubstituted lattice volume, $V_W$ is the effective radii of W in a 6-coordinate environment, $V_V$ is the effective radii of V also in a 6-coordinate environment, and x is the fractional amount of W within the lattice. The resulting strain energies with the corresponding 95% confidence interval based on error propagation for the average structure, the local structure (1.50 Å $\le \, r \,\le$ 19.2 Å) from PDF, the intermediate structure (19.2 Å $\le \, r \,\le$ 30.0 Å) from PDF, and the theoretical strain energy (Fig. 2) demonstrate a significantly higher localized strain as well as increased strain overall compared to the theoretical calculations. These localized strains have been previously suggested as nucleation centers in thin-film^96^ and single-crystal^36^ VO$_2$ systems.

The PDF data shows an increasing probability of finding V−O as the structure becomes more symmetric with increasing W-substitution demonstrated by the increasing peak intensities of the V−O peaks. The V−V interatomic distance also increases from 2.65 Å to 2.92 Å across the W-substitution series demonstrated by the peak maxima shifting with increasing W concentration (Fig. S6). The V–V distance quickly increases at a rate of 0.07(1) Å/at% when W-substitution is below 3.6 at% but this rate decreases by an order of magnitude at higher substitution amounts (3.6–15 at%) (Table S22). This is reminiscent of the fast increase in the *c*/*a* ratio locally followed by a sharp decrease. This increase in the V−V interatomic distance happens similarly in Cr$_x$V$_{1-x}$O$_2$ but conversely Sc-substitution decreases the V−V interatomic distance (Fig. S6).

For the Cr-substitution system, the V−V interatomic distance increases at a rate of 0.010(1) Å/at%. Whereas, for the Sc-substitution system, the V−V interatomic distance decreases at a rate of − 0.0013(9) Å/at%, an order of magnitude lower. The Cr and Sc systems have a significantly slower, almost 1–2 orders of magnitude, respectfully, rate of V−V interatomic distance change (Fig. S6) compared to W-substitution which has a rate of 0.07(1) Å/at% for similar substitution ranges. Phase purity of the Cr- and Sc- substitution systems was verified through Rietveld refinement of synchrotron XRD (Fig. S7). This corresponds well with the MIT differences between the three systems, where Cr increases the MIT by 3 °C/at%^45,47^, Sc decreases the MIT by − 0.7 °C/at%^40^, and W decreases the MIT by − 28 °C/at%^41,61^. The rate of V−V interatomic distance change correlates well ($R^2 = 0.9286$) with the change in the MIT upon increasing substitution amount of the relevant element. This may indicate that V–V dimerization occurs faster in systems that create a larger MIT change, supporting the Peierls hypothesis of structural distortions driving the MIT^1^. This needs further support through experimentation into oxidation states and electronic correlations, however.

**Figure 3:**
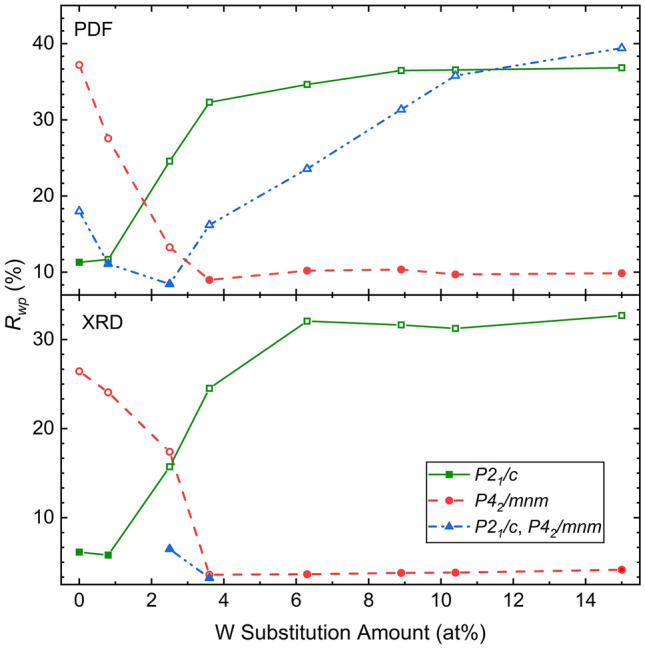
Comparison of the goodness-of-fit parameter, $R_{wp}$, from both XRD (> 30 Å) and PDF data (1.5–30 Å) illustrates how the local structure begins to transition from the monoclinic to the tetragonal structure before the average structure based on tungsten substitution amount. This exemplifies the need for greater local structure studies for predicting and explaining the properties if substituted VO$_2$ is to be engineered and implemented in a functional capacity.

A direct comparison of the residuals from the Rietveld refinement and average real-space Rietveld refinement across the multiple boxcars (Fig. 3) illustrates that the local structure begins transitioning from monoclinic to tetragonal sooner than the average structure based on tungsten-substitution amount. Even after 0.8 at% inclusion of tungsten, locally the structure fits best to a co-refinement to both the monoclinic, and tetragonal structures. But this is not seen until 2.5 at% is reached in the average structure. The local structure is then fully tetragonal at 3.6 at% while the average structure needs to be at 6.3 at% to have a complete phase transformation. This is direct tracking of the nucleation and growth event of the phase transformation.

**Figure 4:**
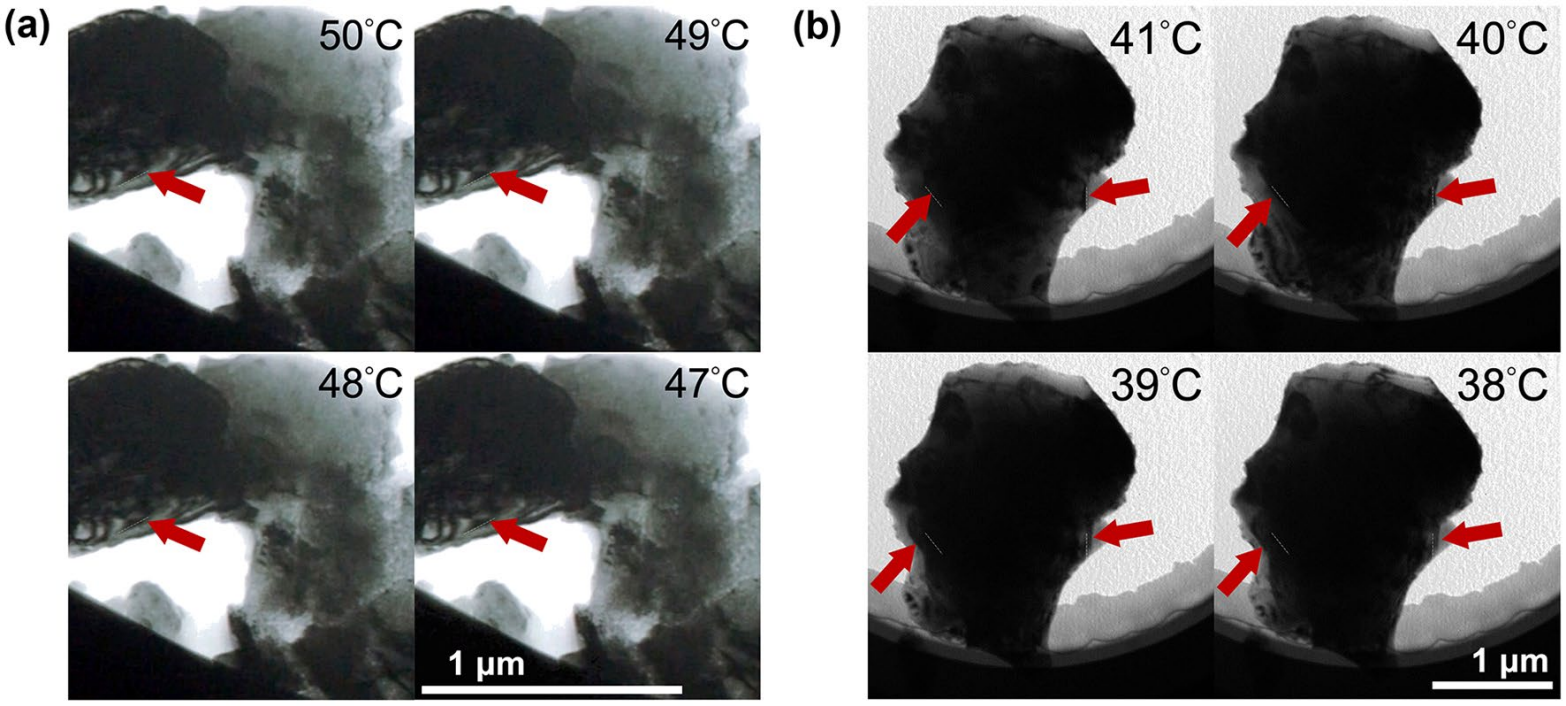
In-situ TEM heating and cooling experiment resulted in structural changes and BF contrast differences allowing for direct observation of the SPT of (**a**) VO$_2$ and (**b**) W$_{0.008}$V$_{0.911}$O$_2$ upon cooling with $T_c$ occurring at 68 °C. The contrast boundary movement, highlighted using red arrows, was used to calculate approximate rates of transformation to compare to in-situ PDF data.

This nucleation and growth is also exhibited through in-situ TEM BF imaging during heating and cooling (Fig. 4). Contrast differences distinguish the high-scattering, lower-symmetry monoclinic phase from the low-scattering, higher-symmetry tetragonal phase. Even at temperatures well below the expected $T_c$ regions of brighter contrast can be seen amidst regions of darker contrast for both the unsubstituted VO$_2$ as well as the W$_{0.008}$V$_{0.992}$O$_2$ (Figs. S8 and S9) illustrating that nuclei of $P4_2/mnm$ are already within the structure prior to the MIT.

Using the in-situ imaging data, we also extracted approximate rates of transformation by tracking the contrast, or phase, boundary migration while cooling. This was then compared to the rate of V–V interatomic distance decrease while cooling from in-situ PDF data. The unsubstituted VO$_2$ resulted in phase transformation rates of 14(31) Å$/^{\circ }$C, and 21(11) Å$/^{\circ }$C for TEM and PDF, respectfully, upon cooling. The 0.8 at% W-substitution gave phase transformation rates of 64(67) Å/$^{\circ }$C and 82(21) Å/$^{\circ }$C for TEM and PDF, respectfully, upon cooling. The large errors are due to the large temperature steps taken during TEM cooling (1 °C) and PDF (2 °C). However, upon comparison of the two samples, the W-substituted phase transformation rate is significantly greater than pure VO$_2$. This suggests W-substitution promotes the local SPT depression.

In-situ PDF’s during heating and cooling were refined similarly to room-temperature PDF. The full *r*-range was used to construct the phase diagram and the boxcar fits were used to track the local lattice parameters (Fig. S5). These results (Figs. S10–S13) were used to validate the accuracy of the unsupervised machine learning results and compare to the TEM BF rate of transformation data.

Non-negative matrix factorization (NMF) is an unsupervised machine learning technique previously applied to XRD^97^ and PDF^98^ analyses. It is similar to the principle component analysis (PCA) method^99^ for encompassing a whole as a sum of its parts. However, NMF is different from PCA in that the parts of the whole are more intuitive for positive-valued data than PCA^100^. Mathematically, NMF decomposes a compressed data set into non-negative components whereas, PCA utilizes an orthogonality constraint.

NMF approximates an $n\, \times \, m$ data set matrix, *V*, by two non-negative matrices *W* ($n\,\times \,r$) and *H* ($r\,\times \,m$), $V \approx WH$. In this in-situ PDF case, NMF was used to cluster the full *r*-range, 30 Å  PDF into pre-transition and post-transition regions. In the case of in-situ PDF, *m* is the number of NMF components used to form the model, *n* the number of data sets which in this case corresponds to the number of different temperatures *G*(*r*) data was collected at, and *r*, in the context of NMF, is the number of data points in each *G*(*r*). Usually the number of components chosen, *m*, is less than both *m* and *r*, compressing the data set. After compressing the data set, the unsupervised part of NMF quantifies the quality of the approximation by calculating a cost function^101^. This tracks the divergence of the data from a linear mixing of the two end members of the data set^97^. The minimization of this deviation was taken as the SPT transition temperature.

The coefficients of linear mixing of these two components tracked across the temperature gradient, resulted in a sigmoidal curve which was a measure of how closely the two NMF-determined components matched the data against the temperature series. These approximately relate to the amount of the component; therefore, the coefficient of linear mixing for component one was normalized between 0 and 100 for simplicity. The inflection point was tracked through the first derivative, and the peak maximum was taken as the transition temperature (Fig. S14). The NMF used the full *r*-range of the data (1.5–30 Å) instead of broken down into boxcars as was done when fitting. This did not significantly change the results from the fitting where the local SPT occurred prior to the long-range SPT.

**Figure 5:**
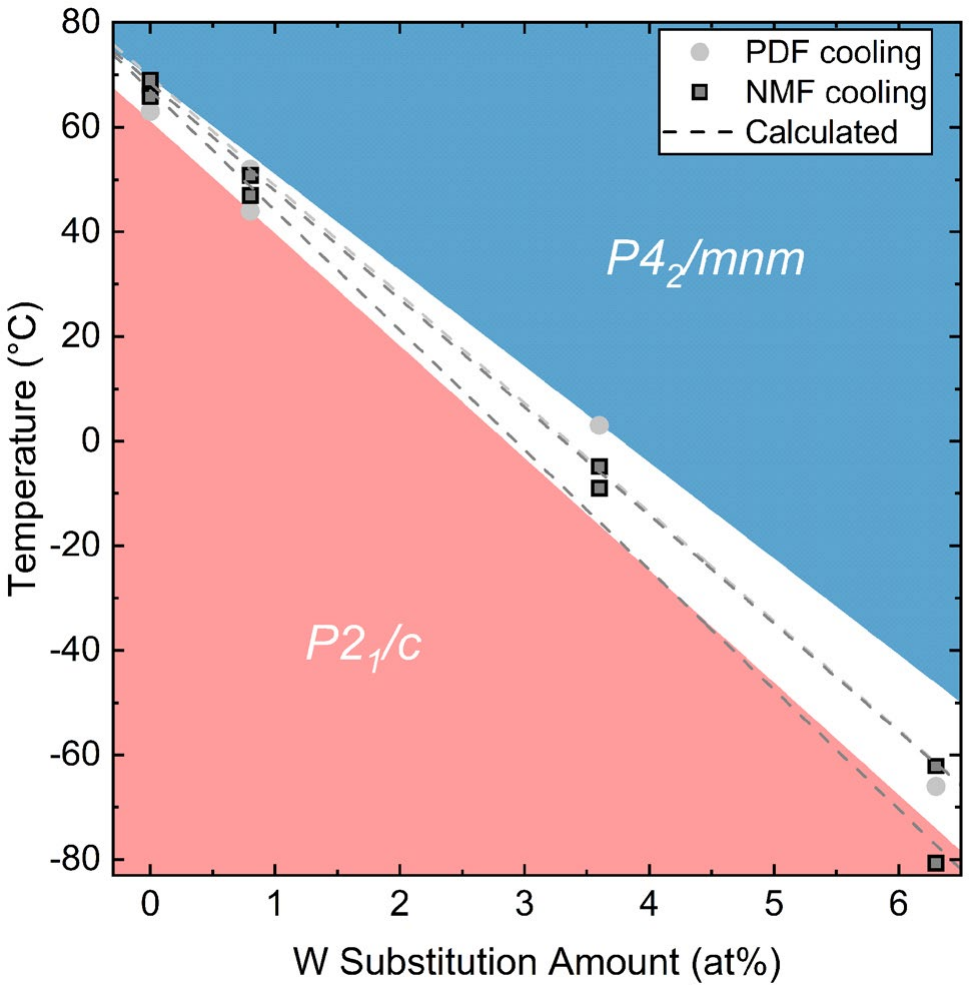
In-situ total scattering distribution refinements elucidated the local phase diagram for the W-substituted VO$_2$ system. These observations were supported through NMF analysis of both the heating and cooling data sets. Linear regressions of each data set, agree with each other and show a slower local progression of the SPT compared to the MIT.

**Figure 6:**
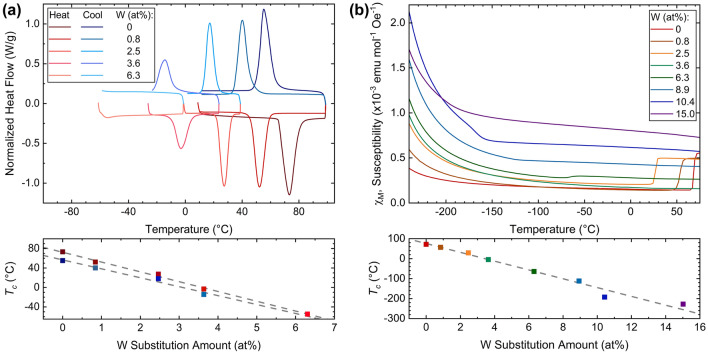
DSC and SQUID magnetization experiments determined the MIT for the tungsten substitution series. The two data sets agree with each other in the rates of $T_c$ depression despite DSC containing a partial data set compared to SQUID. This $T_c$ depression rate also agrees with the structure phase transformation rate from in-situ PDF during heating and cooling.

From the in-situ PDF refinements, a phase diagram (Fig. 5) was produced that shows deviations between the local SPT temperature compared to the average SPT temperature supporting the findings from the room-temperature data that a nucleation and growth phase transformation occurs as observed by others^75^. The rates of $P2_1/c \rightarrow P2_1/c + P4_2/mnm$ structural phase transformation rate was found to be $-23(1) \, ^{\circ }$C/at% given by the NMF analysis. However, a phase transition temperature could not be extracted from in-situ PDF. The rate of structural phase transformation between the $P2_1/c + P4_2/mnm \rightarrow P4_2/mnm$ phases was found to be $-21(1) \, ^{\circ }$C/at% and $-20.7(2) \, ^{\circ }$C/at% for the PDF fitting and NMF analysis, respectively. Further comparison to the bulk properties (Fig. 6) confirms the local SPT not only occurs prior to the bulk SPT but also prior to the bulk MIT. The local SPT happening prior to the bulk SPT and the bulk MIT is critical support of the Peierls transformation occurring prior to the Mott-Hubbard mechanism as hypothesized in c-DMFT studies^69^ and observed by previous characterization techniques^75^.

### Local SPT occurs prior to the bulk MIT

The bulk MIT was characterized using DSC and magnetization experiments (Fig. 6). There is expected hysteresis^37,93,102^ in the heating and cooling $T_c$’s. $T_c$ was determined from the peak maxima positions of the DSC data, and the inflection point of the magnetization data. To determine the inflection point when the W-substitution was greater than 2.5 at%, fitting the Curie-Weiss law ($\chi = \frac{C}{T+\theta _W}+\chi _0$) to the low-temperature ($-200\,^{\circ }$C to $-263\,^{\circ }$C) magnetic susceptibility data was performed (Fig. S15 and Table S33). This fitting was extrapolated to the full temperature range, and subtracted from the overall magnetic susceptibility to emphasize the inflection point at $T_c$.

Magnetization experiments showed that below the MIT, the system follows Curie-Weiss paramagnetism, and after the MIT it follows Pauli paramagnetism and is temperature independent. A similar study on the V$_{1-x}$Mo$_x$O$_2$ system has been performed^58^. Holman et. al. indicated that the substitution of Mo into the system introduces the Curie-Weiss moment seen at temperatures below $-175 \, ^{\circ }$C, which can be extended to the W-substitution system as well. The Weiss temperatures were low, within approximately 10 °C for all samples, demonstrating weak interactions among the magnetic species similar to the Mo-system^58^. The correction factor $\chi _0$ is also very small, on the order of 10$^{-5}$, for all samples. Previous literature found similar results for the Weiss temperature in the W-system^3^.

Using the SPT’s gleaned from refinements of the short-range PDF and long-range PDF data as well as the NMF analysis, linear regressions proffered the similarities between the two techniques and the difference when compared against the rate of MIT. All linear regressions fit well producing $R^2$ values and negative correlation coefficients of $\pm \,0.99$ or greater (Table S34).

The NMF asymmetric Gaussian, correlates to the observed MIT phenomenon. For example, in the DSC (Fig. 6a) the full-width half maximum also increases from 7.95(2) to 8.46(3) with increasing substitution amount from 0 at% to 3.6 at%, respectively. The peak intensity decreases as well, also mimicking the NMF-derived SPT behavior. Also, in comparison to the magnetization data (Fig. 6b) the magnetization differences between the pre-MIT and post-MIT decreases as W substitution amount increases, similar to how the changes in heat flow and SPT magnitude decreases upon increasing W-substitution.

Disagreement between the SPT and MIT can been seen in comparison of the transition temperature depression upon cooling in DSC, − 18(2) °C/at%, and from magnetization experiments, − 22.046(3) °C/at%. The magnetization experiment MIT $T_c$ agrees with the $P2_1/c \rightarrow P2_1/c + P4_2/mnm$ SPT, $-23(1) \, ^{\circ }$C/at%. However, the DSC MIT $T_c$ agrees better with the $P2_1/c + P4_2/mnm \rightarrow P4_2/mnm$ SPT’s, $-21(1) \, ^{\circ }$C/at% and $-20.7(2) \, ^{\circ }$C/at% for the PDF fitting and NMF analysis, respectively. Since magnetization experiments are more sensitive than DSC measurements, it is not necessarily surprising to observe this.

### Localized vanadium oxidation

**Figure 7:**
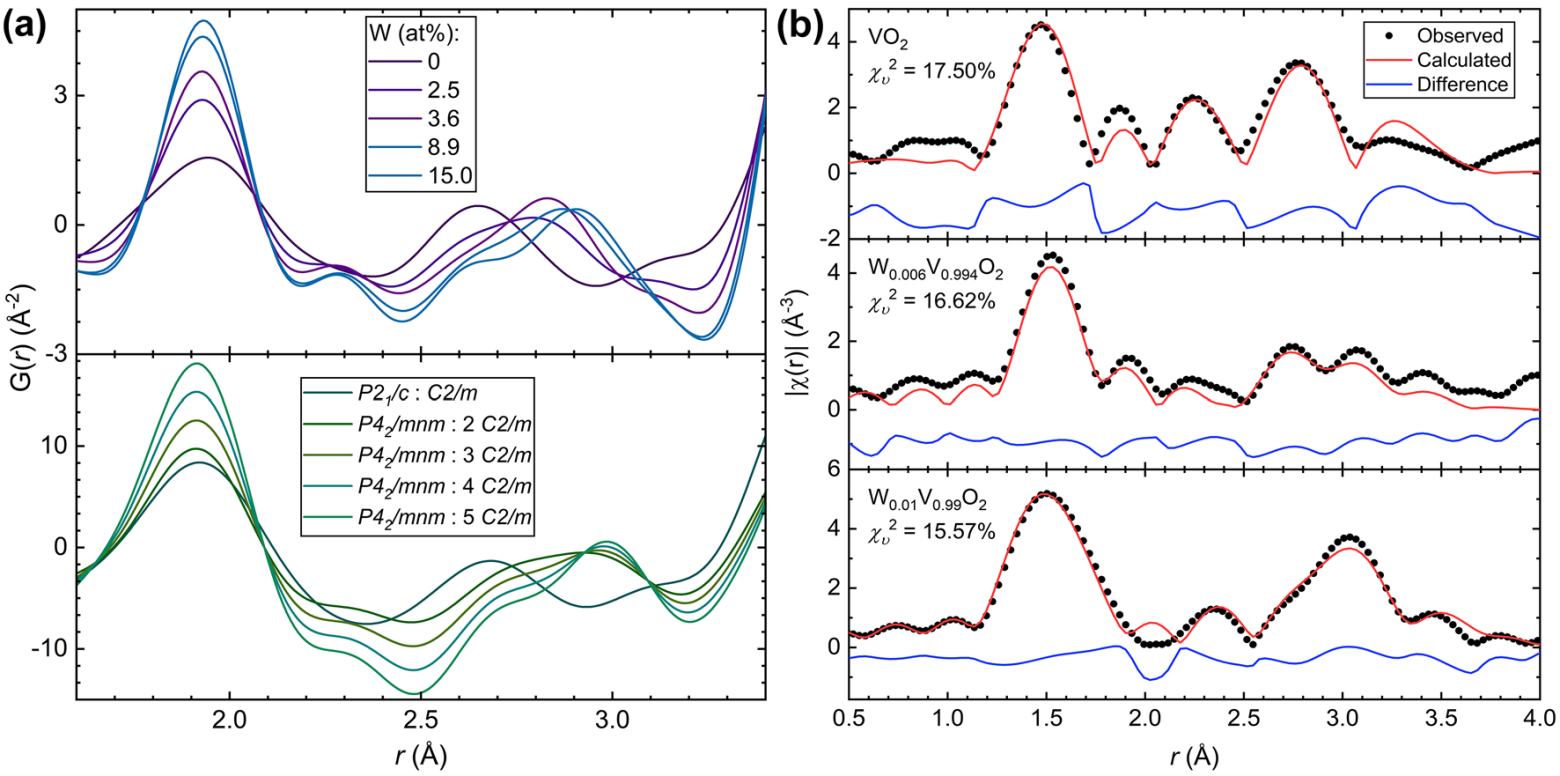
V oxidation is correlated with W-substitution as supported by (**a**) PDF simulations of varying amounts of V$_6$O$_{13}$ (*C*2/*m*) to either VO$_2$ ($P2_1/c$) or VO$_2$ ($P4_2/mnm$) capture the local features of the PDF upon increasing W substitution amount and (Alliance of Bioversity International and CIAT—Multifunctional Landscapes) V *K*-edge EXAFS fitting to paths from the following structures: VO$_2$ ($P2_1/c$), V$_6$O$_{13}$ (*C*2/*m*), V$_2$O$_5$ (*Pnma*), and in the case of the W-substituted samples VO$_2$ ($P2_1/c$), V$_6$O$_{13}$ (*C*2/*m*), V$_2$O$_5$ (*Pnma*), and VO$_2$ ($P4_2/mnm$).

Locally, 1.5–3.6 Å  as W-substitution increased, three unidentified peaks emerged, while $R_wp$ stayed below 10%. By comparing these unidentified local structure peaks between 1.5 and 3.5 Å to simulated PDF patterns, it was deduced that V is undergoing oxidation upon W-substitution. In the V$_6$O$_{13}$ structure, V is in the $4+$ and $5+$ oxidation states. The best simulated representation of the data corresponded to increasing amounts of V$_6$O$_{13}$ in the *C*2/*m* phase along with the main VO$_2$ phase (Fig. 7a) captured by the XRD pattern. As the W-substitution amount increases, the ratio of V$_6$O$_{13}$ (*C*2/*m*) to the main VO$_2$ phase is best described as a linear relationship from the PDF simulation data (Fig. S16). This was corroborated (Table S35) by EXAFS fitting of thin-film W$_x$V$_{1-x}$O$_2$ (Fig. 7b).

According to the Ellingham diagram, V will oxidize W to form V$_2$O$_5$ and WO$_2$ or WO$_3$^103^. Due to the low-substitution amounts, neither crystalline WO$_2$ or WO$_3$ can be seen in the PDF or XRD. But, it seems that there is an intermediate in this redox process is V$_6$O$_{13}$. This oxidation process of VO$_2$ has previously been demonstrated in hydrothermal VO$_2$ syntheses^104–106^ but has not yet been captured in bulk. A potential mechanism for V oxidation is O diffusion through WO$_x$ species. The enhanced O diffusivity in WO$_3$^107^ could provide a pathway for V oxidation in this system.

Refined room-temperature EXAFS data of the V *K*-edge of thin-film W$_x$V$_{1-x}$O$_2$(x = 0, 0.006, and 0.01) gave similar results to the PDF simulations. The best fits were determined through minimizing the goodness-of-fit parameter, $\chi _{\nu }^2$. All three samples refined best to a combination of paths from VO$_2$ ($P2_1/c$), V$_6$O$_{13}$ (*C*2/*m*), V$_2$O$_5$ (*Pnma*), and in the case of all but the unsubstituted sample, VO$_2$ ($P4_2/mnm$) was also needed along with the previous phases to produce the best fit (Table S36). This further supports the oxidation pathway VO$_2 \rightarrow$ V$_6$O$_{13} \rightarrow$ V$_2$O$_5$. These findings support that the local SPT occurs prior to the average MIT.

It was found previously from magnetic susceptibility experiments that W-substitution forms bonding pairs of V$^{3+}$– W$^{6+}$, V$^{3+}$– V$^{4+}$. This was deduced by comparing the effective magnetic moment to a theoretical effective magnetic moment derived from $S_1 = 1$ and $S_2 = \frac{1}{2}$^3^. Using the Curie constant found from fitting, the effective magnetic moment was calculated from $\mu _{eff} = (\frac{3k_B C}{N})^{1/2} \mu _B$ where $k_B$ is Boltzmann’s constant, *C* is the Curie constant from fitting, and *N* is the amount of W-ions in moles, and $\mu _B$ is the Bohr magneton. The theoretical effective magnetic moment was calculated using $\mu _{eff} = g \mu _B [S(S+1)]^{1/2}$ with *g* as the Landé *g*-factor taken as two arising from solely spin contributions to the angular orbital momentum^108^. The number of unpaired electrons, *n* can be approximated by the spin-only magnetic moment, $\mu _{SO} = \mu _{eff} = \sqrt{n(n+2)}$ (Table 1).

**Table 1:** Experimental $\mu _{eff}$ values provided insight into the number of unpaired spins, *n* in the system.

W (at%)	Experimental $\mu _{eff}$ of W ($\mu _B$)	n
0	–	–
0.8	4.42	3.53
2.5	3.17	2.32
3.6	2.73	1.91
6.3	2.30	1.51
8.9	2.47	1.66
10.4	2.83	2.00
15.0	1.63	0.91

Given that the number of unpaired spins is decreasing as the W-substitution amount is increasing, the conclusion is drawn that oxidation is occurring of V and/or W. Adding the results from the PDF and EXAFS fitting, the most likely scenario is the W reducing from $6+$ to $4+$ while the V is oxidizing from $4+$ to $5+$, supported by the Ellingham diagram^103^.

## Conclusions

A W-substitution series of VO$_2$ was analyzed through a slew of diffraction analysis techniques and compared to bulk property measurements from DSC and magnetization. Commonly used average structure identification is not enough to fully capture the complex first-order phase transformation occurring. XRD indeed indicates phase purity and the expected phase transformation, it does a poor job of capturing the local structure though. This local structure is crucial to determine the driving mechanism of this phase transition. More robust analysis of the local structure of phase transitions is needed to comprehensively discuss the origin of these fascinating transformations and ultimately predict them.

XRD indicated phase purity, as well as average lattice expansion upon substitution. The local structure transformation occurs more gradually and prior to the average structure phase transformation. This has not been studied before in regards to the W$_x$V$_{1-x}$O$_2$ system. The PDF structural phase transformation was analyzed through conventional real-space Rietveld fitting techniques as well as NMF modeling. The NMF analysis was able to expediently extract the same and more information as the fitting analysis. PDF fitting was unable to uncover the short-range SPT of highly W-substituted ($> \, 3.6$ at%) samples, but NMF was able to extract this information. NMF analyses of other complex phase transitions would be beneficial to demonstrate differences in the local structure with less direct user involvement. Caution should always be taken when using these techniques as to avoid incorrect conclusions from the data but that does not draw away from the power of more machine based data analysis techniques.

It was found that the average structural phase transformation temperature correlates well with the bulk property metal-insulator transition, with all $T_c$ depression rates being approximately − 20 °C/at% agreeing with previous literature^1^. The local structural phase transformation temperature however, occurs prior to the MIT and average SPT, as illustrated both through fitting of room-temperature PDF, fitting of in-situ PDF, and NMF analysis of the in-situ PDF. This supports the Peierls-Mott hypothesis of the origin of the MIT as proposed by other in-situ experiments^75^, as well as cluster dynamical mean field theory calculations^69^. Thin-film EXAFS fitting and inspection of highly local (1.5 Å- 3.6 Å) PDF data as well as Curie-Weiss fitting of the magnetic susceptibility indirectly uncovered V oxidation due to W-substitution.

## Methods

### Bulk powder synthesis

Reduction of V$_2$O$_5$ to V$_2$O$_3$ was performed under of flow of 5% H$_2$/95% N$_2$ at 800 °C for 24 h. Molar equivalents of V$_2$O$_3$, V$_2$O$_5$, and WO$_2$ or Cr powder or Sc$_2$O$_3$ were then dry-ground for 15 min in an agate mortar and pestle, and sealed under vacuum using conventional Schlenk line techniques into a 9 mm inner diameter fused silica ampoule (mass loading $\approx$ 330 mg. Ampoules were then loaded into a Thermolyne FD1540M Box Furnace equipped with a Eurotherm 2614 temperature control unit and annealed at 1050 °C for 216 h (9 days) with a heating rate of 2 °C/min and a cooling rate of 15 °C/min, except for the Sc-substituted system which was annealed at 1050 °C for 264 h (11 days) with a heating rate of 4 °C/min and a cooling rate of 15 °C/min.

### Thin film synthesis

The procedure for synthesis of VO$_x$ used for generating VO$_2$ thin films was reported by Paik et al.^109^. In brief, oleic acid, 1-octadecene, and dopants were evacuated at 100 °C for 30 min using a Schlenk line technique for purging and refilling with N$_2$. After degassing, the solution was exposed to air and the temperature was increased to 200 °C. At this point, VOCl$_3$ was injected and the solution was aged for an additional 20 min. VO_x_ nanocrystals were collected, washed with ethanol, and centrifuged at 7000 RPM to remove the supernatant. After centrifugation, supernatant was discarded, and nanoparticles were redispersed in hexanes. The dispersion was then dropcast onto 2 cm diameter quartz discs spinning at around 1000 rpm until a homogenous coating was formed. W-substituted VO$_x$ thin films were rapidly thermally annealed to crystalline thin films in a MILA-5000 series RTA (Advance Riko Inc.) at 500 °C for 5 min after the chamber was evacuated to < 1 mTorr.

### Inductively coupled plasma optical emission spectroscopy

Inductively Coupled Plasma Optical Emission Spectroscopy (ICP-OES) was executed using a Perkin Elmer Optima 4300DV spectrometer equipped with a Meinhard concentric glass nebulizer. Samples were digested in a 1:1 mixture of 1% HF:HNO$_3$ for 5 min at 85 °C. Calibration was performed prior to the experiments through a linear regression using standards of varying W (0–15 ppm) and V (30–50 ppm) concentrations. Standards were prepared by diluting commercial stock solutions of 1000 ppm W and V. Tungsten wavelengths analyzed were: 207.912 nm, 224.876 nm, 239.708 nm, and 248.923 nm. Vanadium wavelengths analyzed were: 290.880 nm, 310.230 nm, 309.310 nm, and 292.402 nm.

### Differential scanning calorimetry and magnetization

Differential Scanning Calorimetery (DSC) was conducted on a TA Instruments Discovery DSC 2500 under a heating and cooling rate of 10 °C/min. Magnetization measurements were performed using a Quantum Design MPMS3 at the Ohio State University Nanosystems Lab with an applied magnetic field of 70 kOe and a cooling rate of 10 °C/min. The sample was packed into a pill capsule, and loaded into a straw to avoid aberrant signal. Centering of the capsule was performed prior to the experiment. Thermal equilibration of the sample at each temperature was ensured prior to the collection of data. Fitting parameters to the Curie-Weiss law included the Curie constant, *C*, the Weiss temperature, $\theta _W$, and a correction factor, $\chi _0$ for the temperature independent component of the magnetic susceptibility. The fitting parameters are presented in the supporting information (Table S33). All fitting was completed in OriginPro.

### Synchrotron X-ray diffraction and total scattering

W$_x$V$_{1-x}$O$_2$ and Cr$_x$V$_{1-x}$O$_2$ room-temperature synchrotron x-ray diffraction (XRD) and total scattering pair distribution function (PDF) data was collected at the Advanced Photon Source at Argonne National Laboratory under GUP-68773, GUP-66786, GUP-66783, GUP-62602, GUP-61382 at beamline 17-BM-B ($\lambda$ = 0.24162 Å) at a sample-to-detector distance of 700 mm and 175 mm, respectively. W$_x$V$_{1-x}$O$_2$ variable temperature synchrotron XRD data was collected at the Canadian Light Source at beamline BXDS-WHE ($\lambda$ = 0.3936 Å) at a sample-to-detector distance of 400 mm. Sc$_{x}$V$_{1-x}$O$_2$ syncrotron XRD and PDF data was also collected at the Advanced Photon Source at Argonne National Laboratory at beamline 11-ID-B ($\lambda$ = 0.21150 Å) at sample-to-detector distances of 1000 mm and 180 mm, respectively. In-situ PDF experiments were performed at a sample-to-detector distance of 185 mm to accommodate the Oxford Cryosystems Cryostream 700 Plus situated above the sample capillary. The in-situ experimental configuration is shown in our previous literature^35^. A cooling rate of 6 °C/min preceding and proceeding the phase transformation, and 2 °C/min through the phase transformation ($T_c\, \pm \, 10\,^{\circ }$C) was performed. $T_c$ was determined using DSC and magnetization data prior to the experiments.

Rietveld refinement analysis of the synchrotron XRD data was performed using GSAS-II^78^. The following parameters were refined: (i) lattice parameters, (ii) site occupancies for W (Cr, or Sc) and V, (iii) atomic displacement parameters with the cations held equivalent and the anions held equivalent, (iv) fractional atomic coordinates with W, (Cr, or Sc) and V held equivalent, (v) peak shape, (vi) background Chebyshev coefficients of degree eight, and (vii) scale factor. Refinements utilized the following VO$_2$ CIFs: ICSD-34033 ($P2_1/c$) and ICSD-1504 ($P4_2/mnm$). The site occupancy of the V was altered using VESTA to include the ICP-OES substitution amounts.

All PDF data 2D powder pattern image integration was accomplished using GSAS-II^78^. PDF data reduction and fitting was performed using xPDFsuite^110^. Instrument parameters $Q_{damp}$ and $Q_{broad}$ were obtained through fitting a nickel standard. A Kapton$^{TM}$capillary background was subtracted from the I(q) pattern individually for each sample. W$_x$V$_{1-x}$O$_2$ room-temperature PDF reduction parameters are as follows: $Q_{max-inst} = 21.7$ Å$^{-1}$, $Q_{max} = 21.5$ Å$^{-1}$, $r_{poly} = 0.90$, $Q_{min} = 1.0$ Å$^{-1}$, $Q_{damp} = 0.0086$ Å$^{-1}$, and $Q_{broad} = 0.0274$ Å$^{-1}$. In-situ PDF reduction parameters are as follows: $Q_{max-inst} = Q_{max} = 21$ Å$^{-1}$, $r_{poly} = 0.74$ (except $r_{poly} = 0.90$ for W$_{0.063}$V$_{0.937}$O$_2$), $Q_{min} = 1.0$ Å$^{-1}$, $Q_{damp} = 0.0069$ Å$^{-1}$, and $Q_{broad} = 0.0262$ Å$^{-1}$. Cr$_x$V$_{1-x}$O$_2$ room-temperature PDF reduction parameters are as follows: $Q_{max-inst} = 21.1$ Å$^{-1}$, $Q_{max} = 21.0$ Å$^{-1}$, $r_{poly} = 0.90$, $Q_{min} = 0.7$ Å$^{-1}$, $Q_{damp} = 0.0086$ Å$^{-1}$, and $Q_{broad} = 0.0274$ Å$^{-1}$. Sc$_x$V$_{1-x}$O$_2$ room-temperature PDF reduction parameters are as follows: $Q_{max-inst} = 23.9$ Å$^{-1}$, $Q_{max} = 22.8$ Å$^{-1}$, $r_{poly} = 0.90$, $Q_{min} = 1.1$ Å$^{-1}$, $Q_{damp} = 0.0069$ Å$^{-1}$, and $Q_{broad} = 0.0262$ Å$^{-1}$. The following PDF parameters were refined: (i) scale factor, (ii) lattice parameters, (iii) the quadratic correlation factor, and (iv) atomic displacement parameters $U_{11}$, $U_{22}$, $U_{33}$ using an in-house Python code based on the PDFfit2 code^111^. The PDF data was fit using the following V$_x$O$_y$ .cif’s: ICSD-15028 (V$_6$O$_{13}$, *C*2/*m*), ICSD-254183 (VO$_2$, *C*2/*m*), ICSD-34033 (VO$_2$, $P2_1/c$, and ICSD-1504 (VO$_2$, $P4_2/mnm$). The site occupancy was edited similar to the Rietveld refinements.

### Extended X-ray absorption and fine structure

X-ray absorption spectroscopy experiments were carried out at 20-BM-B and 12-BM-B at the Advanced Photon Source under GUP-34284, GUP-41749. Incident x-ray beam was tuned using a Si(111) double crystal fixed exit monochromator and higher-order harmonics rejected was achieved with a coated mirror. Absorption through thin film samples was measured in focused-beam mode from V *K*-edge. Calibration was performed for V K-edge (5.46376 keV) using V foil^112^. Integration and step sizes for energy ranges are provided in Supporting Information Table S37.

The V *K*-edge EXAFS data was normalized in ATHENA^113^. For all samples the ionization energy was set to 5482.03 eV, and $R_{bkg}$ to 1.2 Å. Ṫhe pre-edge range was $-150$ to $-30$ eV, the normalization order was three, and the normalization range was, 150–866.446 eV. The spline clamps were strong for both the low- and high-energy data.

The normalized EXAFS data was then refined in ARTEMIS^113^. All refinements occurred over the *r*-range 1.2–3.6 Å. Ṫhe following parameters were refined: (i) $E_{not}$, a correctional energy shift, (ii) $S_0^2$, the electronic core-hole relaxation was kept equal for all paths given that it depends on the core element which in this case was V, (iii) phase fraction which was represented as a coefficient to $S_0^2$ and (iv) $\alpha \times R_{eff}$, represented isotropic lattice expansion of the effective interatomic distance from the FEFF calculation Artemis performed. The mean square displacement about the path length, $\sigma ^2$, was not refined as it is contingent upon $S_0^2$ which was already being modified by the phase fraction coefficient.

The phase fraction coefficient was constrained to be between 0.001 and 1.000 for each phase, and the sum of all phase fraction coefficients was restrained to be between 0.999 and 1.000. The paths chosen as the best fit were ones that minimized $\chi _{\nu }^2$ while maintaining − 10 eV $\le \,E_{not}\,\le$ 10 eV, and $S_0^2 \approx 0.7$. The Fourier transform range was chosen based off of Ifeffit’s suggestion, and k weights two and three were fit for all data sets. The following .cif’s were used in FEFF for path generation: ICSD-34033 (VO$_2$, $P2_1/c$), ICSD-1504 (VO$_2$, $P4_2/mnm$), ICSD-15028 (V$_6$O$_{13}$, *C*/2*m*), and ICSD-267175 (V$_2$O$_5$, *Pnma*). For structures with more than one V-site, V$_6$O$_{13}$ and V$_2$O$_5$, the structure was aggregated with FEFF prior to path generation. The final fitting parameter results can be found in Supplemental Table S36.

### Transmission electron microscopy

The BF TEM imaging was collected using a FEI Tecnai F20 TEM, and the heating experiment was performed using a DENSsolutions MEMS-based Wildfire heating holder with heating/cooling rate of 2 °C/min. Powder samples were prepared by dispersal in ethanol and drop-cast onto the electron-transparent windows silicon nitride, SiN$_x$, in the MEMS-based device. Particles directly attached the edge of the electron transparent windows were chosen to ensure temperature homogeneity across the whole particle.

### Non-negative matrix factorization analysis

The dimensionality reduction with non-negative matrix factorization was done using an in-house Python code compiled in Jupyter notebook. The dimensionality of the data is reduced into a 2D space using scikitlearn’s NMF module in the decomposition learning class^114^. Dimensionality was reduced to two components, which were compared to the *G*(*r*) at each temperature. The first derivative was taken of the sigmoid produced when analyzing the linear coefficient of one of the components as a function of temperature. The resulting Gaussian peaks were fit to an asymmetric BiGaussian using OriginPro. The width on either side of the peak half-maximum was fit and the resulting temperature was used as the local SPT or the SPT-onset temperature and the long-range SPT or the SPT-termination temperature.

## Supplementary Information

Supplementary Information.

## Data Availability

W$_x$V$_{1-x}$O$_2$ XRD and ex situ PDF data generated, collected, and processed during this work are available at the Crystallography Open Database 3000344–3000356, and 3000362–3000370, respectively. Cr$_x$V$_{1-x}$O$_2$ and Sc$_x$V$_{1-x}$O$_2$ XRD data generated, collected, and processed during this work are also available at the Crystallography Open Database 3000382–3000384, and 3000375–3000381, respectively. All other data are available from the corresponding author on reasonable request.
