# Supplementary material for: Local structure elucidation of tungsten-substituted vanadium dioxide (V1-xWxO2)
Source: Sci Rep. 2022 Aug 30;12:14767. doi: 10.1038/s41598-022-18575-0 (PMC9428210; doi:10.1038/s41598-022-18575-0)
Supplement: Supplementary file 1 — Supplementary Information. [file 41598_2022_18575_MOESM1_ESM.pdf]

Local structure elucidation of tungsten-substituted vanadium dioxide ($V_{1-x}W_xO_2$)

Catrina E. Wilson,[†] Amanda E. Gibson,[†] Paul M. Cuillier,[†] Cheng-Han Li,[†] Patrice H. N. Crosby,[‡,⊥] Edward B. Trigg,[¶,#] Stan Najmr,[§] Christopher B. Murray,[‖] Joerg R. Jinschek,[†,@] and Vicky Doan-Nguyen[*,†]

†*Ohio State University, Materials Science and Engineering, Columbus, Ohio, 43212, United States*

‡*Ohio State University, Chemistry, Columbus, Ohio, 43212, United States*

¶*Materials & Manufacturing Directorate, Air Force Research Laboratory, WPAFB, OH 45433, United States*

§*University of Pennsylvania, Chemistry, Philadelphia, Pennsylvania, 19143, United States*

‖*University of Pennsylvania, Materials Science and Chemistry, Philadelphia, Pennsylvania, 19143, United States*

⊥*Current address: Cornell University, Human Centered Design, Ithaca, New York, 14853, United States*

#*Current address: Battelle Memorial Institute, Columbus, Ohio, 43201, United States*

@*Current address: Technical University of Denmark, DTU Nanolab, Kongens Lyngby, 2800, Denmark*

E-mail: doan-nguyen.1@osu.edu

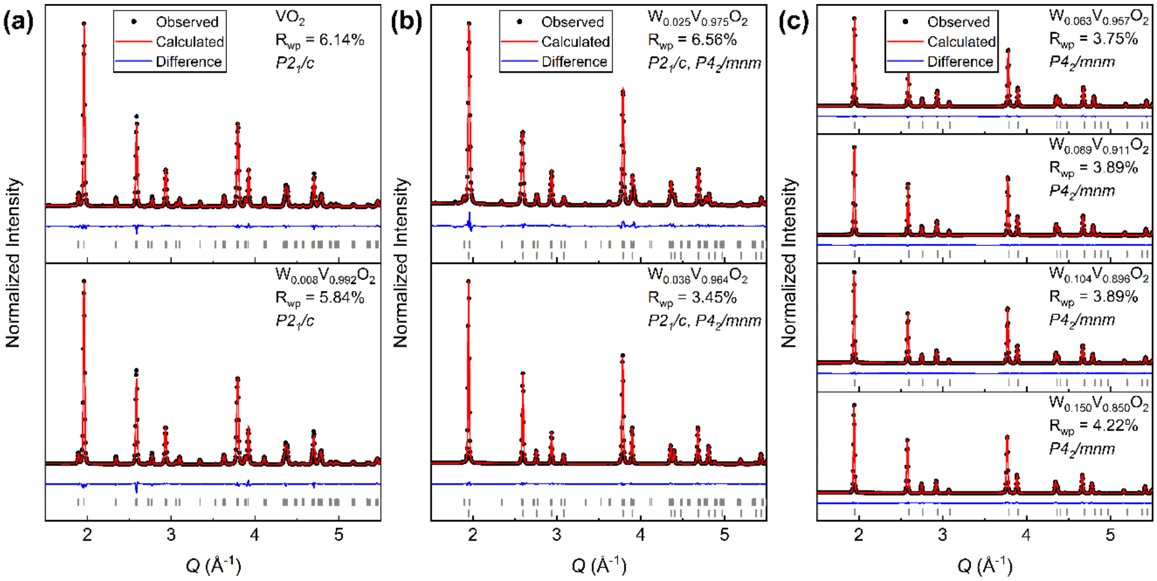

Figure S1: Phase purity and lattice expansion was determined through Rietveld refinement to the (a) $P2_1/c$ (b) $P2_1/c$ and $P4_2/mnm$ (c) and $P4_2/mnm$ phases. The goodness-of-fit value, R_{wp}, was sufficiently low enough (<10%) to validate the accuracy of the model to the data.

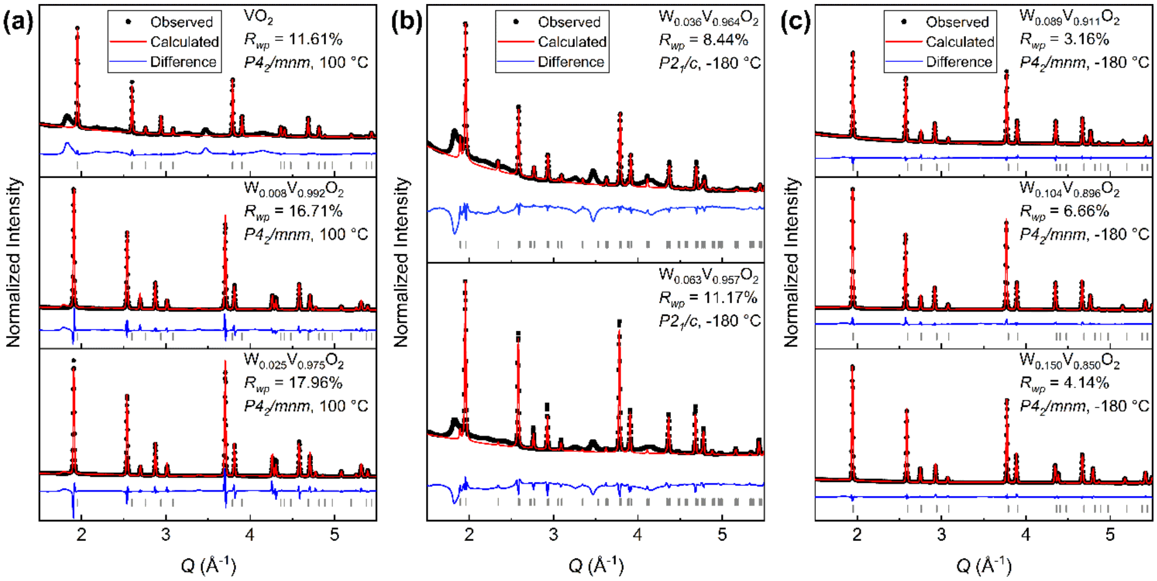

Figure S2: Phase purity and lattice expansion was corroborated through Rietveld refinement of XRD at (a) elevated temperature to $P4_2/mnm$ or (b) depressed temperature to$P2_1/c$ or (c) depressed temperature to $P4_2/mnm$ where the SPT was inaccessible evenat depressed temperature. The broad peaks at 1.8 Å^{-1} and 3.5 Å^{-1} are hypothesized to be due to oxidation of the samples during prolonged storage at ambient conditions.

Table S1: ICP-OES determined precise elemental amounts of substituted tungsten foraccurate fitting models of the powder diffraction data.

W (at%)	Chemical Formula
0	VO_2
0.842(2)	$W_{0.00842(2)}V_{0.99158(2)}O_2$
2.465(2)	$W_{0.02465(4)}V_{0.97535(4)}O_2$
3.635(7)	$W_{0.03635(7)}V_{0.9635(7)}O_2$
6.305(8)	$W_{0.06305(8)}V_{0.93695(8)}O_2$
8.935(6)	$W_{0.08935(6)}V_{0.91065(6)}O_2$
10.430(0)	$W_{0.10430(0)}V_{0.89570(0)}O_2$
15.020(2)	$W_{0.15020(2)}V_{0.84980(2)}O_2$

Table S2: VO_2, $P2_1/c$, structural parameters derived from room-temperature and elevated-temperature synchrotron XRD Rietveld refinement

25 °C, $P2_1/c$:
a = 5.752(2) Å, b = 4.525(2) Å, c = 5.382(2) Å, β = 122.611(4) °, V = 118.022(7) Å^3

Atom	Wyckoff Position	x	y	z	Occ.	U_{iso}(Å^2)
V1	4e	0.2389(3)	0.9797(3)	0.0261(3)	1.000	0.0036(3)
O1	4e	0.105(1)	0.212(1)	0.206(1)	1.000	0.0066(5)
O2	4e	0.406(1)	0.707(1)	0.302(1)	1.000	0.0066(5)

100 °C, $P4_2/mnm$:
a = b = 4.5544(4) Å, c = 2.8526(2) Å, V = 59.17(1) Å^3

Atom	Wyckoff Position	x	y	z	Occ.	U_{iso}(Å^2)
V1	4e	0.00000	0.00000	0.00000	1.000	0.011(1)
O1	4e	0.299(1)	0.299(1)	0.00000	1.000	0.016(3)

Table S3: $W_{0.008}V_{0.992}O_2$, *$P2_1/c$*, structural parameters derived from room-temperature and elevated-temperature synchrotron XRD Rietveld refinement

25 °C, *$P2_1/c$*:
a = 5.757(3) Å, b = 4.5287(2) Å, c = 5.384(2) Å, β = 122.587(5) °, V = 118.262(7) Å^3

Atom	Wyckoff Position	x	y	z	Occ.	U_{iso}(Å^2)
V1	4e	0.2398(2)	0.9810(3)	0.0254(3)	0.991(1)	0.0060(3)
W1	4e	0.2398(2)	0.9810(3)	0.0254(3)	0.009(1)	0.0060(3)
O1	4e	0.106(1)	0.211(1)	0.206(1)	1.000	0.0084(4)
O2	4e	0.405(1)	0.705(1)	0.302(1)	1.000	0.0084(4)

100 °C, *$P4_2/mnm$*:
$a = b$ = 4.6609(3) Å, c = 2.9187(2) Å, V = 64.406(9) Å^3

Atom	Wyckoff Position	x	y	z	Occ.	U_{iso}(Å^2)
V1	4e	0.00000	0.00000	0.00000	0.999(5)	0.01(1)
W1	4e	0.00000	0.00000	0.00000	0.001(5)	0.01(1)
O1	4e	0.2989(9)	0.2989(9)	0.00000	1.000	0.021(2)

.

Table S4: $W_{0.025}V_{0.975}O_2$, $P2_1/c$ and $P4_2/mnm$, structural parameters derived from room-temperature and elevated-temperature synchrotron XRD Rietveld refinement

25°C, $P2_1/c$: Fraction = 0.31(2),
a = 5.766(9) Å, b = 4.5317(5) Å, c = 5.384(8) Å, β = 122.64(1) °, V = 118.45(1) Å^3

Atom	Wyckoff Position	x	y	Z	Occ.	U_{iso}(Å^2)
V1	4e	0.2421(8)	0.985(1)	0.0270(1)	0.971(9)	0.0150(1)
W1	4e	0.2421(8)	0.985(1)	0.0270(1)	0.029(9)	0.0150(1)
O1	4e	0.108(4)	0.207(3)	0.196(4)	1.000	0.017(4)
O2	4e	0.401(4)	0.705(3)	0.298(4)	1.000	0.017(4)

25 °C, $P4_2/mnm$: Fraction = 0.69(2),
$a = b$ = 4.5549(2) Å, c = 2.8542(1) Å, V = 59.216(6) Å^3

Atom	Wyckoff Position	x	y	Z	Occ.	U_{iso}(Å^2)
V1	4e	0.00000	0.00000	0.00000	0.976(5)	0.0074(7)
W1	4e	0.00000	0.00000	0.00000	0.024(5)	0.0074(7)
O1	4e	0.2989(6)	0.2989(6)	0.00000	1.000	0.001(2)

100 °C, $P4_2/mnm$:
$a = b$ = 4.6597(3) Å, c = 2.9188(2) Å, V = 63.375(9) Å^3

Atom	Wyckoff Position	x	y	Z	Occ.	U_{iso}(Å^2)
V1	4e	0.00000	0.00000	0.00000	0.988(4)	0.0048(7)
W1	4e	0.00000	0.00000	0.00000	0.012(4)	0.0048(7)
O1	4e	0.2972(9)	0.2972(9)	0.00000	1.000	0.012(2)

Table S5: $W_{0.036}V_{0.964}O_2$, $P2_1/c$ and $P4_2/mnm$, structural parameters derived from room-temperature and depressed-temperature synchrotron XRD Rietveld refinement

25 °C, $P2_1/c$: Fraction = 0.016(7),
a = 5.75(5) Å, b = 4.587(3) Å, c = 5.38(5) Å, β = 122.59(9) °, V = 119.5(1) Å^3

Atom	Wyckoff Position	x	y	Z	Occ.	U_{iso}(Å^2)
V1	4e	0.2421(8)	0.226(6)	0.995(9)	0.015(6)	0.9(1)
W1	4e	0.2421(8)	0.226(6)	0.995(9)	0.015(6)	0.1(1)
O1	4e	0.108(4)	0.24(2)	0.21(2)	0.28(2)	1.000
O2	4e	0.401(4)	0.36(2)	0.70(2)	0.30(2)	1.000

25 °C, $P4_2/mnm$: Fraction = 0.984(7),
$a = b$ = 4.55883(3) Å, c = 2.85682(4) Å, V = 59.373(2) Å^3

Atom	Wyckoff Position	x	y	Z	Occ.	U_{iso}(Å^2)
V1	4e	0.00000	0.00000	0.00000	0.966(1)	0.0088(2)
W1	4e	0.00000	0.00000	0.00000	0.034(1)	0.0088(2)
O1	4e	0.2996(2)	0.2996(2)	0.00000	1.000	0.0067(5)

-180 °C, $P2_1/c$:
a = 5.75(2) Å, b = 4.539(2) Å, c = 5.38(2) Å, β = 122.47(4) °, V = 118.64(4) Å^3

Atom	Wyckoff Position	x	y	Z	Occ.	U_{iso}(Å^2)
V1	4e	0.234(2)	1.004(4)	0.024(2)	0.966(5)	0.009(2)
W1	4e	0.234(2)	1.004(4)	0.024(2)	0.034(5)	0.009(2)
O1	4e	0.08(1)	0.16(1)	0.189(0)	1.000	0.02(4)
O2	4e	0.395(9)	0.75(1)	0.280(9)	1.000	0.02(4)

Table S6: $W_{0.063}V_{0.937}O_2$, *$P4_2/mnm$*, structural parameters derived from room-temperature and depressed-temperature synchrotron XRD Rietveld refinement

25 °C, $P4_2/mnm$:
$a = b = 4.56276(5)$ Å, $c = 2.86304(3)$ Å, $V = 59.605(2)$ Å^3

Atom	Wyckoff Position	x	y	z	Occ.	U_{iso}(Å^2)
V1	4e	0.00000	0.00000	0.00000	0.937(1)	0.0084(2)
W1	4e	0.00000	0.00000	0.00000	0.063(1)	0.0084(2)
O1	4e	0.2989(2)	0.2989(2)	0.00000	1.000	0.0040(3)

-180 °C, $P2_1/c$:
$a = 5.76(4)$ Å, $b = 4.550(2)$ Å, $c = 5.39(3)$ Å, $\beta = 122.35(6)$ °, $V = 119.32(2)$ Å^3

Atom	Wyckoff Position	x	y	z	Occ.	U_{iso}(Å^2)
V1	4e	0.241(2)	1.003(5)	0.013(2)	0.945(9)	0.006(1)
W1	4e	0.241(2)	1.003(5)	0.013(2)	0.055(9)	0.006(1)
O1	4e	0.10(2)	0.18(1)	0.20(1)	1.000	0.005
O2	4e	0.39(2)	0.74(1)	0.29(1)	1.000	0.005

Table S7: $W_{0.089}V_{0.911}O_2$, *$P4_2/mnm$*, structural parameters derived from room-temperature and depressed-temperature synchrotron XRD Rietveld refinement

25 °C, $P4_2/mnm$:
$a = b = 4.56606(6)$ Å, $c = 2.86885(3)$ Å, $V = 59.812(2)$ Å^3

Atom	Wyckoff Position	x	y	z	Occ.	U_{iso}(Å^2)
V1	4e	0.00000	0.00000	0.00000	0.911(1)	0.0087(2)
W1	4e	0.00000	0.00000	0.00000	0.089(1)	0.0087(2)
O1	4e	0.2988(2)	0.2988(2)	0.00000	1.000	0.0024(4)

-180 °C, $P4_2/mnm$:
$a = b = 4.56124(8)$ Å, $c = 2.88795(6)$ Å, $V = 60.084(3)$ Å^3

Atom	Wyckoff Position	x	y	z	Occ.	U_{iso}(Å^2)
V1	4e	0.00000	0.00000	0.00000	0.923(1)	0.006(5)
W1	4e	0.00000	0.00000	0.00000	0.077(1)	0.006(5)
O1	4e	0.2928(4)	0.2928(4)	0.00000	1.000	0.005

Table S8: $W_{0.104}V_{0.896}O_2$, $P4_2/mnm$, structural parameters derived from room-temperature and depressed-temperature synchrotron XRD Rietveld refinement

25 °C, $P4_2/mnm$:
$a = b = 4.56954(6)$ Å, $c = 2.87232(4)$ Å, $V = 59.976(2)$ Å^3

Atom	Wyckoff Position	x	y	z	Occ.	U_{iso}(Å^2)
V1	4e	0.00000	0.00000	0.00000	0.897(1)	0.0095(2)
W1	4e	0.00000	0.00000	0.00000	0.103(1)	0.0095(2)
O1	4e	0.2984(2)	0.2984(2)	0.00000	1.000	0.0028(4)

-180 °C, $P4_2/mnm$:
$a = b = 4.56631(9)$ Å, $c = 2.88937(6)$ Å, $V = 60.247(3)$ Å^3

Atom	Wyckoff Position	x	y	z	Occ.	U_{iso}(Å^2)
V1	4e	0.00000	0.00000	0.00000	0.905(2)	0.01(3)
W1	4e	0.00000	0.00000	0.00000	0.095(2)	0.03(7)
O1	4e	0.2952(4)	0.2952(4)	0.00000	1.000	0.0090(7)

Table S9: $W_{0.150}V_{0.850}O_2$, $P4_2/mnm$, structural parameters derived from room-temperature and depressed-temperature synchrotron XRD Rietveld refinement

25 °C, $P4_2/mnm$:
$a = b = 4.57272(6)$ Å, $c = 2.87862(4)$ Å, $V = 60.191(2)$ Å^3

Atom	Wyckoff Position	x	y	z	Occ.	U_{iso}(Å^2)
V1	4e	0.00000	0.00000	0.00000	0.849(3)	0.0121(2)
W1	4e	0.00000	0.00000	0.00000	0.151(3)	0.0121(2)
O1	4e	0.2978(3)	0.2978(3)	0.00000	1.000	0.0024(8)

-180 °C, $P4_2/mnm$:
$a = b = 4.57222(6)$ Å, $c = 2.86690(4)$ Å, $V = 59.933(2)$ Å^3

Atom	Wyckoff Position	x	y	z	Occ.	U_{iso}(Å^2)
V1	4e	0.00000	0.00000	0.00000	0.865(2)	0.01(1)
W1	4e	0.00000	0.00000	0.00000	0.135(2)	0.01(2)
O1	4e	0.2953(3)	0.2953(3)	0.00000	1.000	0.005

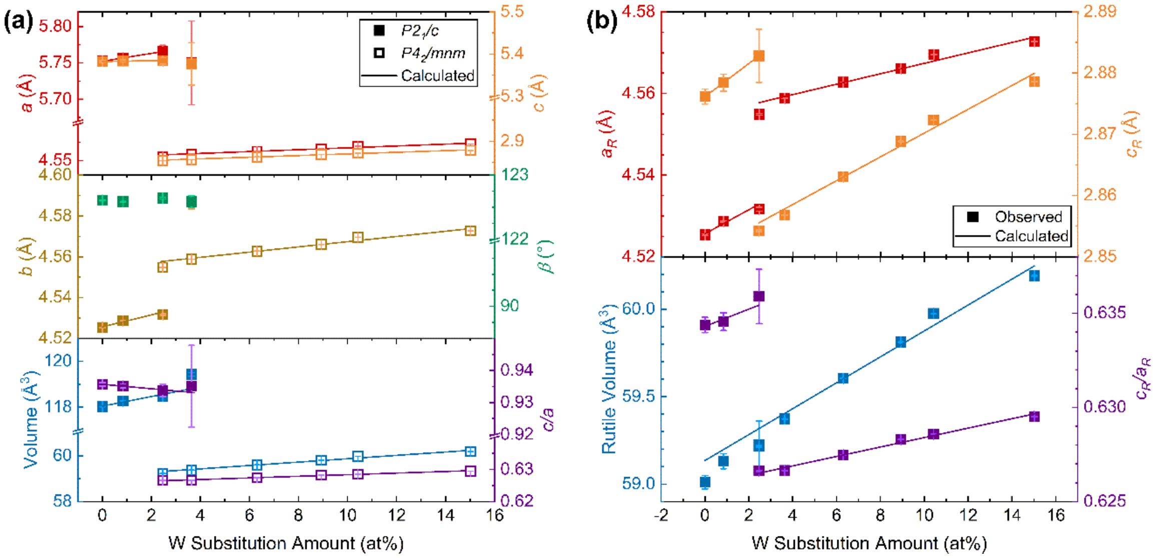

Figure S3: Lattice expansion was tracked as a function of substitution amount basedon the lattice parameters extracted from the Rietveld refinement. (a) The lattice expands along the *a*-, *b*-, and *c*-axes corresponding to an increase in the overall cell volume as low- substitution amounts of W are added while the structure is still in the monoclinic phase. Once the structure transitions to the tetragonal phase, the unit cell expansion continues as expected due to ionic radii differences between V and W. (b) Transformation of all lattice parameters onto the rutile, *P4₂/mnm*, lattice demonstrates the discontinuity at the SPT is due to the first order phase transition and not the change in unit cell definitions.

Table S10: Linear regression parameters of the room-temperature lattice parameters derived from Rietveld refinement across the $W_xV_{1-x}O_2$ series

$P2_1/c$ Lattice Parameters	Number of Points	Degrees of Freedom	Pearson's r	R-Square	Intercept	Slope (at%$^{-1}$)
a (Å)	3	1	0.99997	0.99994	5.75230(3)	0.00547(4)
b (Å)	3	1	0.97017	0.94123	4.5257(6)	0.0030(7)
c (Å)	3	1	0.91839	0.84343	2.851(1)	0.0019(1)
β (°)	3	1	-0.11493	0.97358	122.060(2)	-0.01(3)
V (Å^3)	4	2	0.93839	0.88058	118.05(5)	0.21(5)
c/a	4	2	-0.99328	0.98661	0.93571(4)	-7.1(6)E-4
**$P4_2/mnm$ Lattice Parameters**	**Number of Points**	**Degrees of Freedom**	**Pearson's r**	**R-Square**	**Intercept**	**Slope (at%$^{-1}$)**
$a = b$ (Å)	6	4	0.98056	0.96149	4.555(1)	0.0013(1)
c (Å)	6	4	0.99089	0.98185	2.850(1)	0.0019(1)
V (Å^3)	6	4	0.98857	0.97727	59.14(5)	0.073(6)
c/a	6	4	0.99163	0.98334	0.6259(2)	2.5(2)E-4

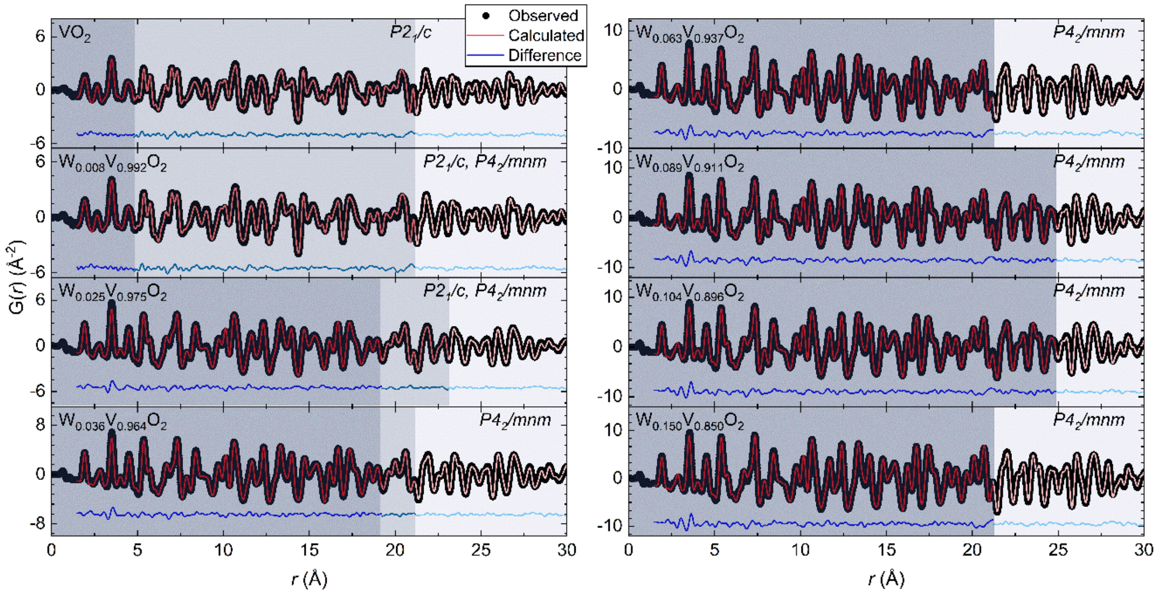

Figure S4: Boxcar analysis of the pair distribution function from to-tal scattering synchrotron diffraction illustrates local phase purity to the phases indicated with all R_{wp} < 10%. Three boxcars were needed for the less-symmetric monoclinic phase fits and as the structure transformed into the more-symmetric tetragonal phase, only two boxcars were needed to accurately capture the structure.

Table S11: PDF refinement parameters of VO_2

$P2_1/c$ Lattice Parameters:	1.50 Å – 4.80 Å	4.80 Å – 21.2 Å	21.2 Å – 29.9 Å
a (Å)	5.68(6)	5.752(4)	5.749(4)
b (Å)	4.55(4)	4.528(3)	4.524(4)
c (Å)	5.34(7)	5.381(4)	5.383(4)
β (°)	122(1)	122.64(5)	122.62(5)
V U_{11} (Å^2)	0.003(4)	0.0023(7)	0.003(2)
V U_{22} (Å^2)	0.001(2)	0.0023(7)	0.003(1)
V U_{33} (Å^2)	0.001(2)	0.0013(5)	0.0018(8)

Table S12: PDF refinement parameters of $W_{0.008}V_{0.992}O_2$

$P2_1/c$ Lattice Parameters:	1.50 Å – 4.80 Å	4.80 Å – 21.2 Å	21.2 Å – 29.9 Å
Scale Factor	0.49(4)	0.44(2)	0.39(2)
a (Å)	5.70(6)	5.759(4)	5.756(4)
b (Å)	4.56(3)	4.535(3)	4.533(4)
c (Å)	5.39(6)	5.386(4)	5.388(4)
β (°)	122(1)	122.64(5)	122.63(5)
V(W) U_{11} (Å^2)	0.002(2)	0.0030(9)	0.004(2)
V(W) U_{22} (Å^2)	0.002(3)	0.0027(7)	0.005(3)
V(W) U_{33} (Å^2)	0.001(3)	0.0030(8)	0.004(2)
$P4_2/mnm$ Lattice Parameters:	1.50 Å – 4.80 Å	4.80 Å – 21.2 Å	21.2 Å – 29.9 Å
Scale Factor	0.04(3)	0.03(1)	0.03(2)
a = *b* (Å)	4.5(2)	4.55(2)	4.53(1)
c (Å)	2.9(3)	2.85(3)	2.89(3)

Table S13: PDF refinement parameters of $W_{0.025}V_{0.975}O_2$

$P2_1/c$ Lattice Parameters:	1.50 Å – 19.2 Å	19.2 Å – 23.1 Å	23.1 Å – 29.9 Å
Scale Factor	0.18(1)	0.05(3)	0.11(3)
a (Å)	5.77(2)	5.75(5)	5.76(1)
b (Å)	4.540(9)	4.55(6)	4.54(1)
c (Å)	5.40(2)	5.39(4)	5.39(2)
β (°)	122.9(2)	122.7(7)	122.7(2)
$P4_2/mnm$ Lattice Parameters:	1.50 Å – 19.2 Å	19.2 Å – 23.1 Å	23.1 Å – 29.9 Å
Scale Factor	0.37(1)	0.40(3)	0.31(2)
a = *b* (Å)	4.554(2)	4.555(3)	4.554(2)
c (Å)	2.859(3)	2.858(3)	2.856(2)
V(W) U_{33} (Å^2)	0.008(1)	0.006(3)	0.005(4)

Table S14: PDF refinement parameters of $W_{0.036}V_{0.963}O_2$

$P4_2/mnm$ Lattice Parameters:	1.50 Å – 19.2 Å	19.2 Å – 21.2 Å	21.2 Å – 29.9 Å
a = *b* (Å)	4.560(1)	4.564(3)	4.5590(9)
c (Å)	2.859(2)	2.857(3)	2.858(1)
V(W) U_{33} (Å^2)	0.0067(6)	0.005(5)	0.006(1)

Table S15: PDF refinement parameters of $W_{0.063}V_{0.957}O_2$

$P4_2/mnm$ Lattice Parameters:	1.50 Å – 21.2 Å	21.2 Å – 29.9 Å
a = *b* (Å)	4.5638(8)	4.5626(7)
c (Å)	2.864(1)	2.864(1)
V(W) U_{33} (Å^2)	0.0064(4)	0.006(1)

Table S16: PDF refinement parameters of $W_{0.089}V_{0.911}O_2$

$P4_2/mnm$ Lattice Parameters:	1.50 Å – 21.2 Å	21.2 Å – 29.9 Å
a = *b* (Å)	4.5677(7)	4.5666(7)
c (Å)	2.870(1)	2.8704(9)
V(W) U_{33} (Å^2)	0.0067(4)	0.0058(8)

Table S17: PDF refinement parameters of $W_{0.104}V_{0.896}O_2$

P4₂/mnm Lattice Parameters:	1.50 Å – 24.9 Å	24.9 Å – 29.9 Å
$a = b$ (Å)	4.5717(4)	4.5720(9)
c (Å)	2.8742(7)	2.874(1)
V(W) U_{33} (Å^2)	0.0068(3)	0.004(1)

Table S18: PDF refinement parameters of $W_{0.150}V_{0.850}O_2$

$P4_2/mnm$ Lattice Parameters:	1.50 Å – 21.2 Å	21.2 Å – 29.9 Å
$a = b$ (Å)	4.5730(7)	4.5722(7)
c (Å)	2.8791(9)	2.8793(9)
V(W) U_{33} (Å^2)	0.0073(4)	0.0059(7)

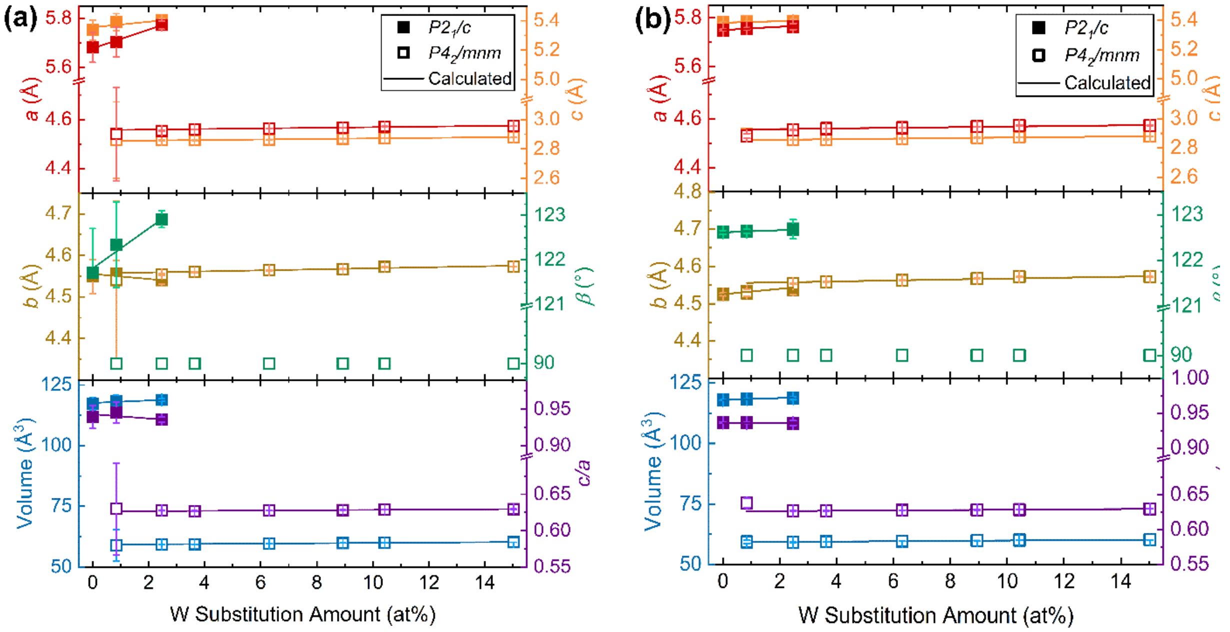

Figure S5: Observation of the lattice parameters based on the boxcar PDF analysis of thePDF shows a larger increase in the lattice parameters in the short-range boxcar than in the long-range boxcar, especially in the monoclinic phase.

Table S19: Rates of lattice parameter change upon W-substitution across different spatial regions of the sample based on PDF refinements.

$P2_1/c$ Lattice Parameter Rates:	0 Å – 19.2 Å	19.2 Å – 30 Å	> 30 Å
a (Å/at%)	0.039(3)	0.007(1)	0.00547(4)
b (Å/at%)	-0.006(3)	0.007(3)	0.0030(7)
c (Å/at%)	0.02(1)	0.0049(9)	0.0011(5)
β (°/at%)	0.45(7)	0.024(5)	-0.01(3)
V (Å^3/at%)	0.5(2)	0.4(1)	0.21(5)
c/*a* (at%$^{-1}$)	-0.003(2)	0.00027(1)	-0.00071(6)
$P4_2/mnm$ Lattice Parameter Rates:	0 Å – 19.2 Å	19.2 Å – 30 Å	> 30 Å
a = *b* (Å/at%)	0.0012(3)	0.0013(2)	0.0013(1)
c (Å/at%)	0.0018(2)	0.0019(1)	0.0019(1)
V (Å^3/at%)	0.070(1)	0.071(7)	0.073(6)
c/*a* (at%$^{-1}$)	0.00022(2)	0.00023(2)	0.00025(2)

Table S20: Short-range linear regression parameters of the room-temperature lattice parameters derived from PDF refinement across the $W_xV_{1-x}O_2$ series

$0 \leq$ W (at%) ≤ 2.5:

$P2_1/c$ Lattice Parameters	Number of Points	Degrees of Freedom	Pearson's *r*	*R*-Square	Intercept	Slope (at%$^{-1}$)
a (Å)	3	1	0.99724	0.99448	5.677(7)	0.039(3)
b (Å)	3	1	-0.88283	0.77938	4.555(7)	-0.006(3)
c (Å)	3	1	0.88408	0.78159	5.4(2)	0.02(1)
β (°)	3	1	0.98958	0.97927	121.8(2)	0.45(7)
V (Å^3)	4	2	0.95762	0.91704	117.6(4)	0.5(2)
c/*a*	4	2	-0.79489	0.63186	0.943(5)	-0.003(2)

$0.8 \leq$ W (at%) ≤ 15.0:

$P4_2/mnm$ Lattice Parameters	Number of Points	Degrees of Freedom	Pearson's *r*	*R*-Square	Intercept	Slope (at%$^{-1}$)
a = *b*(Å)	7	5	0.9117	0.8312	4.556(3)	0.0012(3)
c (Å)	7	5	0.97748	0.95546	2.854(1)	0.0018(2)
V (Å^3)	7	5	0.95523	0.91246	59.2(1)	0.07(1)
c/*a*	7	5	0.97452	0.94968	0.6263(2)	2.2(2)E-4

Table S21: Long-range linear regression parameters of the room-temperature lattice parameters derived from PDF refinement across the $W_xV_{1-x}O_2$ series

$0 \leq$ W (at%) ≤ 2.5:

$P2_1/c$ Lattice Parameters	Number of Points	Degrees of Freedom	Pearson's *r*	*R*-Square	Intercept	Slope (at%$^{-1}$)
a (Å)	3	1	0.98778	0.97570	5.7498(8)	0.007(1)
b (Å)	3	1	0.93946	0.88258	4.525(2)	0.007(3)
c (Å)	3	1	0.98342	0.96711	5.383(5)	0.0049(9)
β (°)	3	1	0.97766	0.95583	122.616(4)	0.024(5)
V (Å^3)	4	2	0.96142	0.92433	117.98(8)	0.4(1)
c/a	4	2	-0.99921	0.99841	0.93626(8)	-2.7(1)E-4

$0.8 \leq$ W (at%) ≤ 15.0:

$P4_2/mnm$ Lattice Parameters	Number of Points	Degrees of Freedom	Pearson's *r*	*R*-Square	Intercept	Slope (at%$^{-1}$)
a = *b*(Å)	7	5	0.92915	0.86332	4.555(2)	0.0013(2)
c (Å)	7	5	0.98991	0.97991	2.852(1)	0.0019(1)
V (Å^3)	7	5	0.97551	0.95161	59.18(7)	0.071(7)
c/a	7	5	0.97608	0.95273	0.6262(2)	2.3(2)E-4

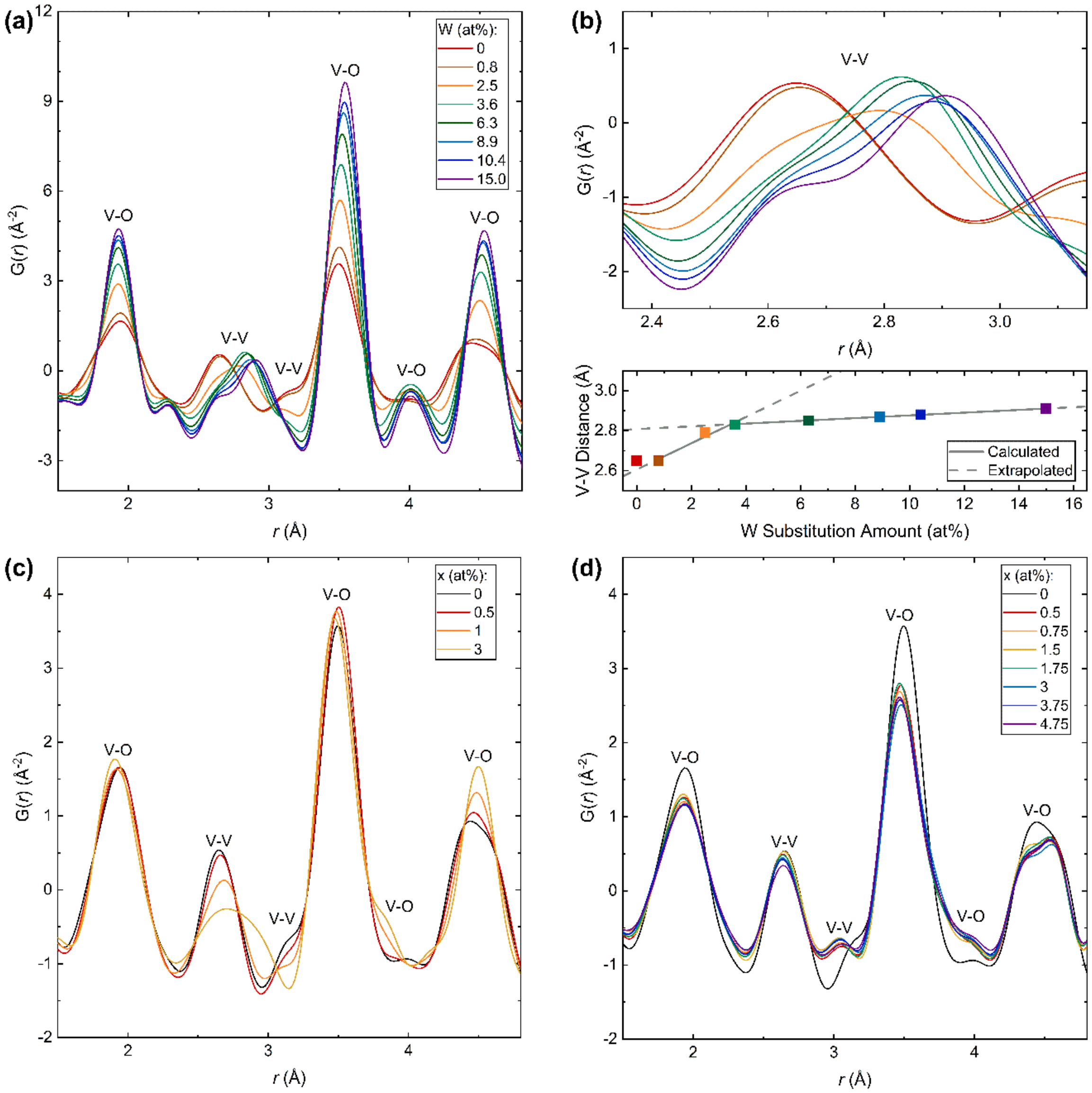

Figure S6: Inspection of the room temperature PDF measurements shows (a) an increase in the V-V atomic distance from 2.65 Å to 2.92 Å. The peak at 3.13 Å also disappears as the structure becomes more symmetric but peaks at 2.28 Å and 2.64 Å also arise upon increasing tungsten substitution (b) tracking the V-V peak shift and comparing to the XRD data illustrates an order of magnitude decrease in the phase transformation rate and comparison to (c) Cr-substitution and (d) Sc-substitution series it seems the V-V interatomic distance rate of change correlates with the MIT rate of change upon increasing substitution.

Table S22: *G(r)* V-V interatomic distance linear regression parameters across the $W_xV_{1-x}O_2$ series demonstrates a better fit to two W-ranges, R^2_{avg} = 0.98172, than to one, R^2 = 0.69765.

Linear Regression W Range (at%)	Number of Points	Degrees of Freedom	Pearson's *r*	*R*-Square	Intercept (Å)	Slope (Å/at%)
0.8 – 15.0	7	5	0.83526	0.69765	2.73(3)	0.014(4)
0.8 – 3.6	3	1	0.98251	0.96533	2.61(3)	0.07(1)
3.6 – 15.0	4	2	0.99906	0.99811	2.808(2)	0.0068(2)

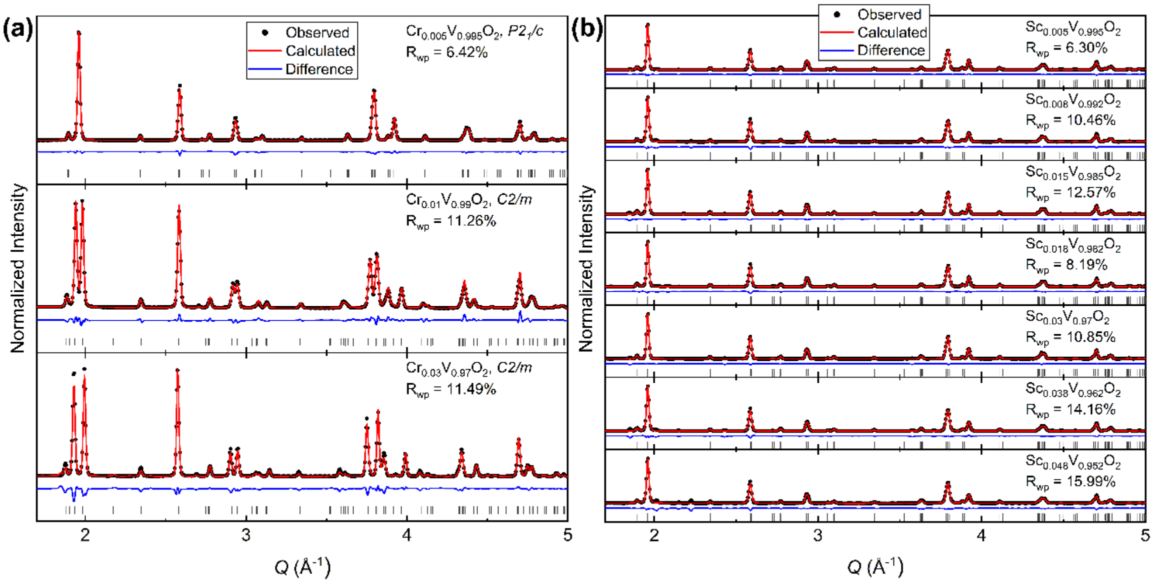

Figure S7: Rietveld refinements of (a) $Cr_xV_{1-x}O_2$ and (b) $Sc_xV_{1-x}O_2$ validates phase purityfor both systems.

Table S23: $Cr_{0.005}V_{0.995}O_2$, $P2_1/c$, structural parameters derived from room-temperature synchrotron XRD Rietveld refinement.

$Cr_{0.005}V_{0.995}O_2$, $P2_1/c$:
a = 5.753(2) Å, b = 4.5267(2) Å, c = 5.382(2) Å, β = 122.583(4)°, V = 118.095(6) Å^3

Atom	Wyckoff Position	x	y	z	Occ.	U_{iso}(Å^2)
V1	4e	0.2386(2)	0.9790(2)	0.0254(2)	0.91(3)	0.0046(2)
Cr1	4e	0.2386(2)	0.9790(2)	0.0254(2)	0.09(3)	0.0046(2)
O1	4e	0.106(1)	0.213(1)	0.206(1)	1.000	0.0069(4)
O2	4e	0.404(1)	0.708(1)	0.303(3)	1.000	0.0069(4)

Table S24: $Cr_{0.01}V_{0.99}O_2$, *C2/m*, structural parameters derived from room-temperature synchrotron XRD Rietveld refinement.

$Cr_{0.01}V_{0.99}O_2$, *C2/m*:
a = 9.064(1) Å, *b* = 5.7682(3) Å, *c* = 4.5236(5) Å, β = 91.203(3)°, *V* = 236.46(3) Å^3

Atom	Wyckoff Position	x	y	z	Occ.	U_{iso}(Å^2)
V1	4g	0.00000	0.7174(4)	0.00000	0.93(6)	0.0053(3)
Cr1	4g	0.00000	0.7174(4)	0.00000	0.07(6)	0.0053(3)
V2	4i	0.2333(4)	0.00000	0.5266(7)	0.93(6)	0.0053(3)
Cr2	4i	0.2333(4)	0.00000	0.5266(7)	0.07(6)	0.0053(3)
O1	8j	0.151(1)	0.246(1)	0.286(3)	1.000	0.0063(8)
O2	4i	0.394(2)	0.00000	0.201(3)	1.000	0.0063(8)
O3	4i	0.102(2)	0.00000	0.794(3)	1.000	0.0063(8)

Table S25: $Cr_{0.03}V_{0.97}O_2$, *C2/m*, structural parameters derived from room-temperature synchrotron XRD Rietveld refinement.

$Cr_{0.03}V_{0.97}O_2$, *C2/m*:
a = 9.0654(7) Å, *b* = 5.7913(2) Å, *c* = 4.5269(4) Å, β = 91.927(2)°, *V* = 237.53(2) Å^3

Atom	Wyckoff Position	x	y	z	Occ.	U_{iso}(Å^2)
V1	4g	0.00000	0.7222(4)	0.00000	0.86(6)	0.0078(5)
Cr1	4g	0.00000	0.7222(4)	0.00000	0.14(6)	0.0078(5)
V2	4i	0.2336(3)	0.00000	0.5275(7)	0.86(6)	0.0078(5)
Cr2	4i	0.2336(3)	0.00000	0.5275(7)	0.14(6)	0.0078(5)
O1	8j	0.149(3)	0.248(1)	0.293(3)	1.000	0.0054(8)
O2	4i	0.392(1)	0.00000	0.199(3)	1.000	0.0054(8)
O3	4i	0.106(1)	0.00000	0.809(3)	1.000	0.0054(8)

Table S26: $Sc_{0.005}V_{0.995}O_2$, *P2₁/c*, structural parameters derived from room-temperature synchrotron XRD Rietveld refinement.

$Sc_{0.005}V_{0.995}O_2$, $P2_1/c$:
a = 5.756(3) Å, *b* = 4.5281(2) Å, *c* = 5.386(2) Å, β = 122.606(4)°, *V* = 118.242(9) Å^3

Atom	Wyckoff Position	x	y	z	Occ.	U_{iso}(Å^2)
V1	4e	0.2384(3)	0.9788(3)	0.0254(3)	0.93(2)	0.0033(4)
Sc1	4e	0.2384(3)	0.9788(3)	0.0254(3)	0.07(2)	0.0033(4)
O1	4e	0.107(1)	0.210(1)	0.208(1)	1.000	0.0093(3)
O2	4e	0.401(1)	0.705(1)	0.301(1)	1.000	0.0093(3)

Table S27: $Sc_{0.0075}V_{0.9925}O_2$, *$P2_1/c$*, structural parameters derived from room-temperature synchrotron XRD Rietveld refinement.

$Sc_{0.0075}V_{0.9925}O_2$, *$P2_1/c$*:
a = 5.756(7) Å, *b* = 4.5277(4) Å, *c* = 5.384(6) Å, β = 122.59(1)°, *V* = 118.21(2) Å^3

Atom	Wyckoff Position	x	y	z	Occ.	U_{iso}(Å^2)
V1	4e	0.2390(7)	0.9789(7)	0.0263(6)	0.92(4)	0.0047(8)
Sc1	4e	0.2390(7)	0.9789(7)	0.0263(6)	0.08(4)	0.0047(8)
O1	4e	0.098(3)	0.218(3)	0.203(3)	1.000	0.009(1)
O2	4e	0.408(3)	0.709(2)	0.308(2)	1.000	0.009(1)

Table S28: $Sc_{0.015}V_{0.985}O_2$, *$P2_1/c$*, structural parameters derived from room-temperature synchrotron XRD Rietveld refinement.

$Sc_{0.015}V_{0.985}O_2$, *$P2_1/c$*:
a = 5.756(6) Å, *b* = 4.5286(4) Å, *c* = 5.386(5) Å, β = 122.62(1)°, *V* = 118.27(2) Å^3

Atom	Wyckoff Position	x	y	z	Occ.	U_{iso}(Å^2)
V1	4e	0.2392(7)	0.9798(7)	0.0268(7)	0.91(5)	0.0027(9)
Sc1	4e	0.2392(7)	0.9798(7)	0.0268(7)	0.09(5)	0.0027(9)
O1	4e	0.102(3)	0.217(3)	0.199(3)	1.000	0.008(1)
O2	4e	0.405(3)	0.710(3)	0.303(3)	1.000	0.008(1)

Table S29: $Sc_{0.0175}V_{0.9825}O_2$, *$P2_1/c$*, structural parameters derived from room-temperature synchrotron XRD Rietveld refinement.

$Sc_{0.0175}V_{0.9825}O_2$, *$P2_1/c$*:
a = 5.754(4) Å, *b* = 4.5271(3) Å, *c* = 5.384(4) Å, β = 122.602(7)°, *V* = 118.15(1) Å^3

Atom	Wyckoff Position	x	y	z	Occ.	U_{iso}(Å^2)
V1	4e	0.2389(4)	0.9792(4)	0.0262(4)	0.94(3)	0.0018(6)
Sc1	4e	0.2389(4)	0.9792(4)	0.0262(4)	0.06(3)	0.0018(6)
O1	4e	0.103(2)	0.212(2)	0.206(2)	1.000	0.0081(8)
O2	4e	0.404(2)	0.709(2)	0.302(2)	1.000	0.0081(8)

Table S30: $Sc_{0.03}V_{0.97}O_2$, *P2₁/c*, structural parameters derived from room-temperature synchrotron XRD Rietveld refinement.

$Sc_{0.03}V_{0.97}O_2$, $P2_1/c$:
a = 5.757(5) Å, b = 4.5285(3) Å, c = 5.387(5) Å, β = 122.613(8)°, V = 118.28(2) Å^3

Atom	Wyckoff Position	x	y	z	Occ.	U_{iso}(Å^2)
V1	4e	0.2393(6)	0.9788(5)	0.0263(6)	0.91(4)	0.0018(7)
Sc1	4e	0.2393(6)	0.9788(5)	0.0263(6)	0.09(4)	0.0018(7)
O1	4e	0.105(5)	0.213(2)	0.202(2)	1.000	0.006(1)
O2	4e	0.404(2)	0.707(2)	0.300(2)	1.000	0.006(1)

Table S31: $Sc_{0.0375}V_{0.9625}O_2$, $P2_1/c$, structural parameters derived from room-temperature synchrotron XRD Rietveld refinement.

$Sc_{0.0375}V_{0.9625}O_2$, $P2_1/c$:
a = 5.756(9) Å, b = 4.5277(6) Å, c = 5.384(8) Å, β = 122.59(2)°, V = 118.22(3) Å^3

Atom	Wyckoff Position	x	y	z	Occ.	U_{iso}(Å^2)
V1	4e	0.2388(8)	0.9797(9)	0.0260(9)	0.91(6)	0.006(1)
Sc1	4e	0.2388(8)	0.9797(9)	0.0260(9)	0.10(6)	0.006(1)
O1	4e	0.100(4)	0.216(3)	0.201(3)	1.000	0.013(2)
O2	4e	0.407(4)	0.709(3)	0.307(3)	1.000	0.013(2)

Table S32: $Sc_{0.0475}V_{0.9525}O_2$, $P2_1/c$, structural parameters derived from room-temperature synchrotron XRD Rietveld refinement.

$Sc_{0.0475}V_{0.9525}O_2$, $P2_1/c$:
a = 5.76(1) Å, b = 4.5281(7) Å, c = 5.38(1) Å, β = 122.58(2)°, V = 118.24(3) Å^3

Atom	Wyckoff Position	x	y	z	Occ.	U_{iso}(Å^2)
V1	4e	0.238(1)	0.977(1)	0.026(1)	0.85(7)	0.004(1)
Sc1	4e	0.238(1)	0.977(1)	0.026(1)	0.15(7)	0.004(1)
O1	4e	0.088(4)	0.225(4)	0.199(3)	1.000	0.008(2)
O2	4e	0.416(5)	0.710(4)	0.315(3)	1.000	0.008(2)

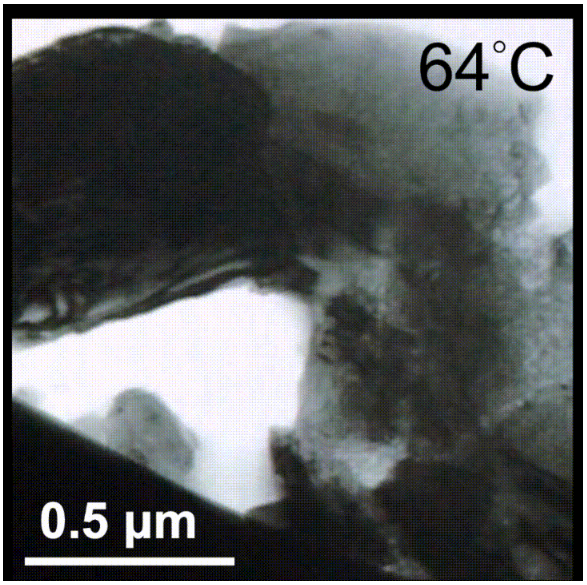

Figure S8: *In-situ* bright-field TEM images of pure VO_2 show the phase boundary movement during cooling and nuclei perpetuation well below T_c.

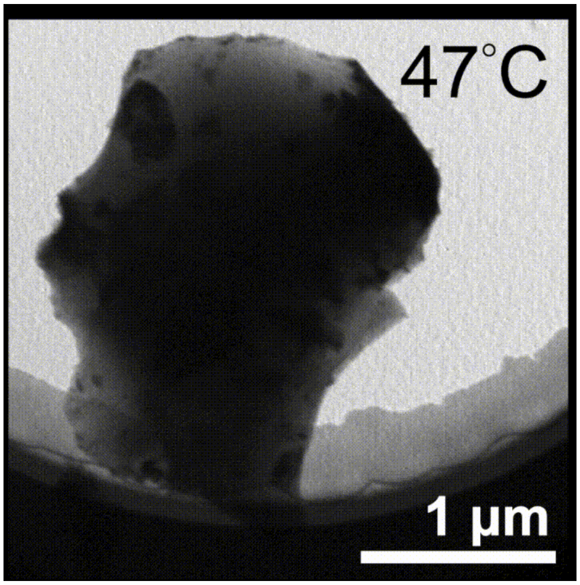

Figure S9: *In-situ* bright-field TEM images of $W_{0.008}V_{0.992}O_2$ show the rapid phase boundary movement during cooling compared to pure VO_2 with nuclei perpetuation well below T_c is maintained.

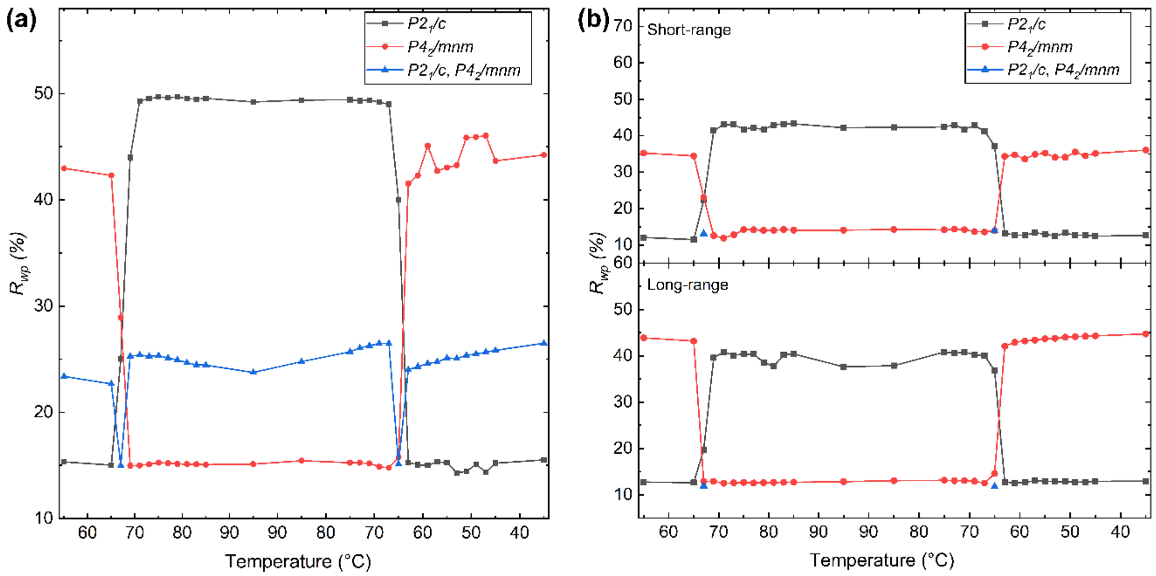

Figure S10: PDF fitting goodness-of-fit parameter, R_{wp}, for in situ heating/cooling of VO_2 (a) across the full r-range (1.5 Å – 30 Å) and (b) across the short-range (1.5 Å – 19.2 Å) and the long-range (19.2 Å – 30 Å)

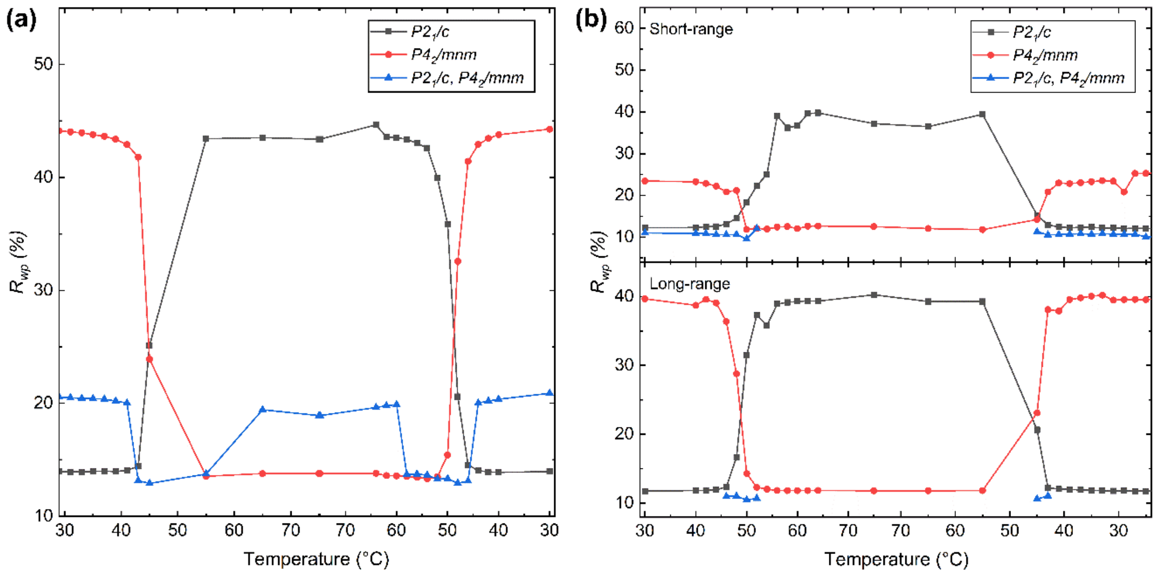

Figure S11: PDF fitting goodness-of-fit parameter, R_{wp}, for in situ heating/cooling of $W_{0.008}V_{0.992}O_2$ (a) across the full r-range (1.5 Å – 30 Å) and (b) across the short-range (1.5 Å – 16.9 Å) and the long-range (16.9 Å – 30 Å)

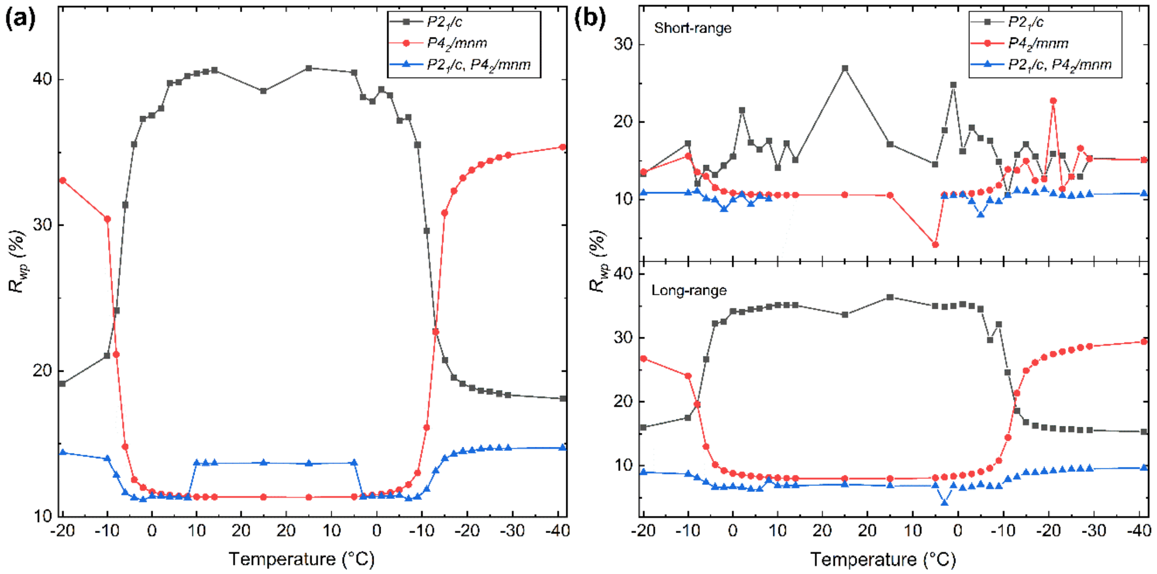

Figure S12: PDF fitting goodness-of-fit parameter, R_{wp}, for in situ heating/cooling of $W_{0.036}V_{0.964}O_2$ (a) across the full r-range (1.5 Å – 30 Å) and (b) across the short-range (1.5 Å – 19.2 Å) and the long-range (19.2 Å – 30 Å)

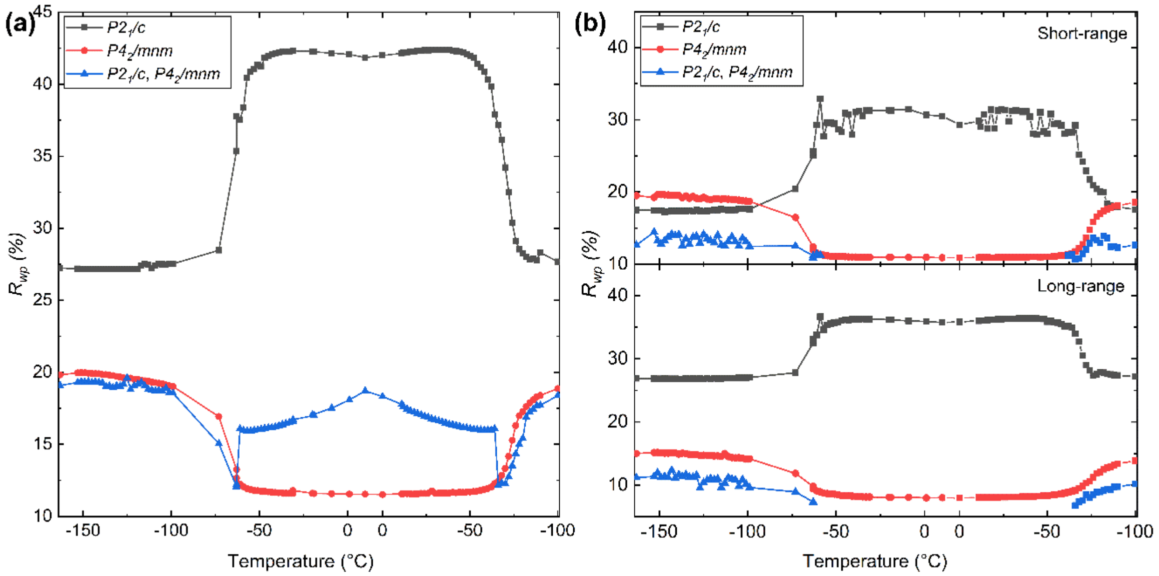

Figure S13: PDF fitting goodness-of-fit parameter, R_{wp}, for in situ heating/cooling of $W_{0.063}V_{0.937}O_2$ (a) across the full r-range (1.5 Å – 30 Å) and (b) across the short-range (1.5 Å – 19.2 Å) and the long-range (19.2 Å – 30 Å)

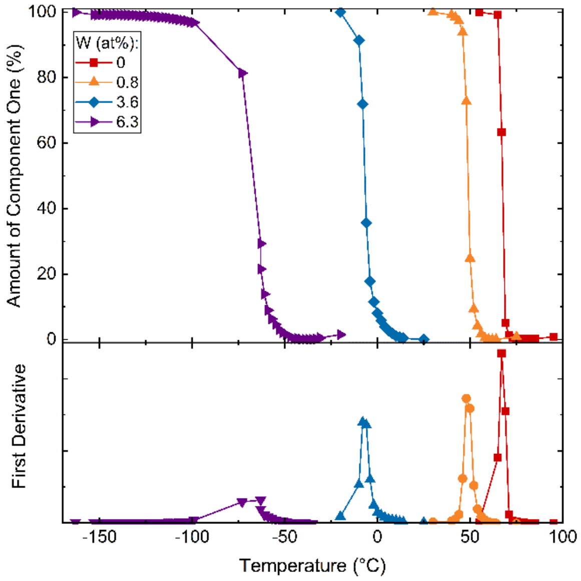

Figure S14: NMF analysis of the *in-situ* heating data revealed that for the substitution amounts less than 0.8 at%, the local transition temperature is lower than the average transition temperature, similar to the fitting analysis. However, upon substitution of 3.6 at% and greater the local transition temperature is lower than the average transition temperature.

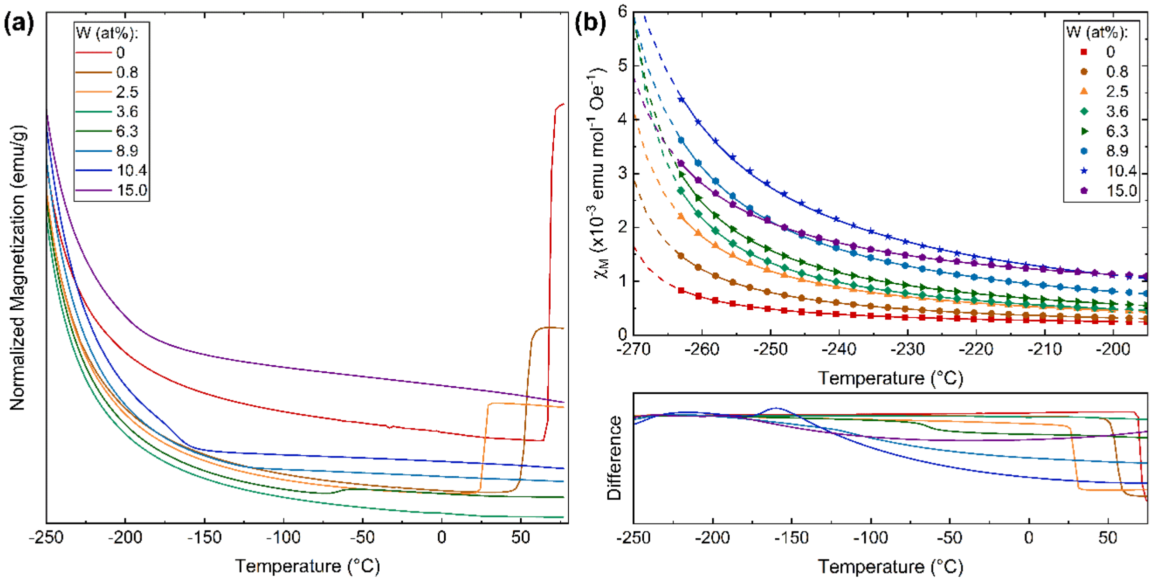

Figure S15: Normalized magnetization data (a) better reflects the decrease in changeof magnetization across the MIT due to the local structure already transitioning to the tetragonal phase. The fits to the Curie-Weiss law subtracted from the data (b) were used to determine the transition temperature of highly substituted samples. This fit was performed over -263°C to $-$ 200°C and extrapolated for the rest of the data.

Table S33: DSC linear regression parameters across the $W_xV_{1-x}O_2$ series ($0 \leq x \leq 0.063$) and magnetization linear regression parameters across the $W_xV_{1-x}O_2$ series ($0 \leq x \leq 0.150$).

DSC	Number of Points	Degrees of Freedom	Pearson's r	R-Square	Intercept (°C)	Slope (°C/at%)
Heating	5	3	-0.9981	0.99621	72(2)	-20.1(7)
Cooling	4	2	-0.98785	0.97585	57(4)	-18(2)

Magnetization	Number of Points	Degrees of Freedom	Pearson's r	R-Square	Intercept (°C)	Slope (°C/at%)
Cooling	8	6	-0.98699	0.97414	70(11)	-21(1)

Table S34: Curie-Weiss fitting parameters of the low-temperature ($-263^{\circ}\mathrm{C} \leq T \leq -200^{\circ}\mathrm{C}$) magnetization data $W_xV_{1-x}O_2$ series ($0 \leq x \leq 0.150$).

W Amount (at%)	C	χ_0 (E-5 emu mol^{-1} Oe^{-1})	θ_w (K)
0	0.00925(6)	0.130(1)	3.09(8)
0.842(2)	0.01952(8)	6.8(1)	3.89(5)
2.465(2)	0.0315(2)	5.4(4)	4.6(1)
3.635(7)	0.03351(7)	4.4(1)	2.69(3)
6.305(8)	0.0417(1)	4.2(2)	4.11(4)
8.935(6)	0.0679(6)	-2.9(7)	8.5(1)
10.430(0)	0.104(2)	-0.1(2)	13.1(3)
15.020(2)	0.0500(4)	0.51(5)	8.7(1)

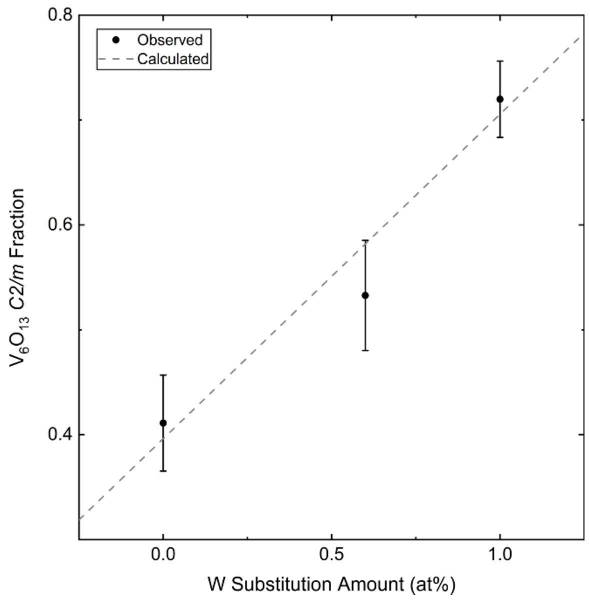

Figure S16: The fraction of V_6O_{13} *C2/m* extracted from EXAFS refinement, where the phase fraction was represented as a coefficient to S_0^2, increases linearly as a function of increasing W-substitution amount.

Table S35: V *K*-edge EXAFS refinement V_6O_{13} linear regression parameters across the thin-film $W_xV_{1-x}O_2$ series

Number of Points	Degrees of Freedom	Pearson's *r*	*R*-Square	Intercept (V_6O_{13} Fraction)	Slope (V_6O_{13} Fraction/at%)
3	1	0.98038	0.96115	0.40(5)	0.31(6)

Table S36: EXAFS refinement parameters over the *r*-range 1.2 Å – 3.6 Å of thin-film $W_xV_{1-x}O_2$ with a Hanning *k*-window over *k*-weights two and three

Parameters	VO_2	$W_{0.006}V_{0.994}O_2$	$W_{0.01}V_{0.99}O_2$
k-range ($Å^{-1}$)	3.0 – 14.05	3.0 – 14.5	3.0 – 14.0
S_0^2	0.75(7)	0.66(6)	1.2(1)
E_{not}	3(2)	5.5(8)	7(1)
α	0.012(4)	0.007(3)	0.014(4)
$n\,(P2_1/c)$	0.25(4)	0.33(5)	0.08(3)
$n\,(P4_2/mnm)$	–	0.089(2)	0.07(2)
$n\,(C2/m)$	0.41(5)	0.53(5)	0.72(4)
$n\,(Pnma)$	0.34(4)	0.052(4)	0.13(2)
**Paths**	VO_2	$W_{0.006}V_{0.994}O_2$	$W_{0.01}V_{0.99}O_2$
	$P2_1/c$ O1.1	$P2_1/c$ O1.3	$P2_1/c$ O2.3
	$P2_1/c$ V1.1	$P2_1/c$ V1.2	$P2_1/c$ V1.1
	$P2_1/c$ V1.2	$P4_2/mnm$ O1.1	$P2_1/c$ V1.2
	$P2_1/c$ V1.3	$P4_2/mnm$ O1.3	$P2_1/c$ V1.6
	$P2_1/c$ V1.4	$P4_2/mnm$ O1.1 V1.2	$P4_2/mnm$ O1.1
	$P2_1/c$ V1.5	*C2/m* O SS 1.648	$P4_2/mnm$ O1.3
	C2/m O SS 1.763	*C2/m* O SS 1.888	$P4_2/mnm$ O1.1 V1.2
	C2/m O SS 1.980	*C2/m* O SS 2.079	*C2/m* O SS 2.079
	C2/m O SS 2.079	*C2/m* V SS 3.047	*Pnma* O SS 1.749
	C2/m V SS 3.275	*C2/m* O SS 3.456	*Pnma* O SS 1.887
	C2/m O SS 3.456	*Pnma* O SS 1.568	*Pnma* O SS 1.968
	C2/m V SS 3.626	*Pnma* O SS 1.749	*Pnma* O SS 2.000
	Pnma O SS 1.568	*Pnma* O SS 2.000	*Pnma* V SS 3.065
	Pnma O SS 1.828	*Pnma* V SS 3.155	*Pnma* V SS 3.155
	Pnma O SS 1.887	*Pnma* V SS 3.586	
	Pnma O SS 2.000		
	Pnma V SS 3.065		

Table S37: V *K*-edge EXAFS experimental conditions.

W Amount (at%)	Energy from Edge (eV)	Step Size (eV)	Integration Time (s)	Weight
0	-150 – -10	5	1	1
0.6	-10 – 40	0.2	1	1
1.0	40 – 971.41	0.05	1	1
